# Suppressing Morphological and Energetic Disorder in Copper Antimony Sulfide‐based Hole‐Transporting Materials via Ligand–Precursor Engineering for Efficient and Stable Perovskite Solar Cells

**DOI:** 10.1002/advs.76977

**Published:** 2026-07-31

**Authors:** Ibrahimhan Dilci, Savas Sonmezoglu

**Affiliations:** ^1^ Department of Metallurgical and Materials Engineering Karamanoglu Mehmetbey University Karaman Türkiye; ^2^ Nanotechnology R&D Laboratory Karamanoglu Mehmetbey University Karaman Türkiye

**Keywords:** copper antimony sulfide nanocrystals, dopant‐free, inorganic hole‐transporting materials, ligand‐coordination chemistry, perovskite solar cells, sulfur‐precursor engineering

## Abstract

Dopant‐free inorganic hole‐transport layers (HTLs) are promising for improving the efficiency and stability of perovskite solar cells (PSCs). Here, CuSbS_2_ nanocrystals are engineered through sulfur‐precursor and ligand‐coordination chemistry using hexamethyldisilathiane (TMS) and thiourea (ThU) combined with oleylamine/oleic acid (OAm/OAc) ligands. While the TMS route reduces platelet dimensions, the ThU precursor with an optimized OAm: OAc ratio of 3:7 suppresses excessive anisotropic growth and induces mixed plate‐like/quasi‐spherical nanostructures, leading to denser particle packing and improved interfacial coverage. Structural and electronic analyses reveal that sulfur‐release kinetics and ligand coordination govern morphology evolution, energetic disorder, and interfacial charge‐transfer behavior. As a result, PSCs employing ThU‐derived CuSbS_2_ HTLs achieve a champion power conversion efficiency of 22.72% with 0.82 for fill factor, outperforming TMS‐derived (20.00%) and ES‐derived (17.31%) counterparts. The optimized devices also exhibit enhanced operational stability under illumination and thermal aging conditions. These findings establish sulfur‐precursor and ligand engineering as an effective strategy for high‐performance inorganic HTLs in PSCs.

## Graphical abstract

Sulfur‐precursor and ligand engineering regulate the nucleation, morphology, and energetic disorder of CuSbS_2_ nanocrystals for dopant‐free inorganic hole‐transport layers in perovskite solar cells. Thiourea‐assisted synthesis combined with optimized OAm/OAc coordination suppresses excessive anisotropic growth, enabling mixed plate‐like/quasi‐spherical nanostructures with improved interfacial coverage and charge transport. The optimized CuSbS_2_ HTL delivers a champion power conversion efficiency of 22.72% together with enhanced operational stability under humidity, thermal, and illumination stress conditions.

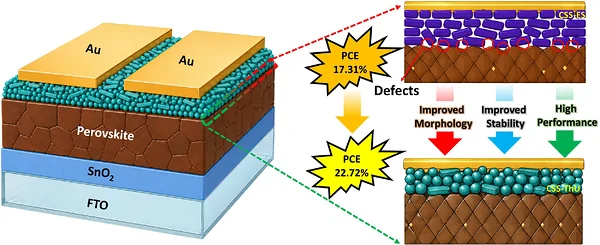

## 1 Introduction

Perovskite solar cells (PSCs) have rapidly advanced as a leading photovoltaic technology due to their exceptional optoelectronic properties and compatibility with low‐temperature solution processing. Since the pioneering report by Miyasaka and co‐workers in 2009, power conversion efficiency (PCE) has dramatically increased from 3.8% to over 28% for single‐junction devices [1, 2]. This progress originates from the intrinsic properties of metal halide perovskites, including strong optical absorption, long carrier diffusion lengths, low exciton binding energies, and defect‐tolerant electronic structures [3, 4]. Despite these advantages, the long‐term stability of PSCs remains a critical challenge, particularly under exposure to moisture, thermal stress, and continuous illumination [5, 6, 7]. From this perspective, a primary strategy is to replace unstable organic hole‐transport materials (HTM) with chemically robust inorganic alternatives in particular for improving device durability. These charge transport layers play a crucial role in both the long‐term stability and performance of the device by enabling not only efficient charge extraction at the perovskite/electrode interface, suppressing interfacial recombination, and ensuring proper energy‐level alignment, but also mitigating defect formation and ion migration at the interface [8, 9, 10, 11]. Although Spiro‐OMeTAD remains the benchmark hole transport layer (HTL) in high‐efficiency PSCs, its low intrinsic conductivity requires dopants such as Li‐TFSI and tert‐butylpyridine, which introduce hygroscopicity, mobile ionic species, and interfacial instability, ultimately accelerating device degradation [12, 13, 14]. These limitations have motivated extensive efforts toward the development of dopant‐free inorganic‐based HTLs with improved chemical and thermal stability. Among them, Cu‐based oxides have been explored in conventional n–i–p PSCs because of their chemical robustness, suitable valence‐band positions, and good thermal stability. However, the application of oxide nanoparticle HTLs in the this architecture can be constrained by relatively low intrinsic hole mobility, limited interparticle electronic coupling, and the difficulty of forming compact and conformal layers on the rough perovskite surface through low‐temperature solution processing [15, 16, 17].

Compared with oxide HTLs, Cu‐based chalcogenides offer several attractive features for solution‐processed inorganic HTLs. The stronger covalent interaction between Cu 3d and S 3p orbitals can give rise to a more dispersive valence band, facilitating efficient hole transport. In addition, sulfur atoms possess a softer Lewis basic character than oxygen, enabling stronger interactions with undercoordinated Pb^2^^+^ ions at the perovskite surface, which can contribute to interfacial defect passivation and suppressed non‐radiative recombination [18, 19]. Furthermore, colloidal chalcogenide nanocrystals can be readily deposited onto perovskite absorbers to form compact and continuous hole‐transport layers under mild processing conditions. Among them, copper antimony sulfides (CuSbS_2_) have emerged as a promising material owing to their suitable valence band position, intrinsic p‐type conductivity, and eco‐friendly composition. Its p‐type character arises mainly from copper vacancies and antisite defects, which introduce shallow acceptor states enabling efficient hole transport [20, 21, 22]. Moreover, the valence band of CuSbS_2_ is well aligned with commonly used perovskite absorbers, allowing for efficient hole extraction and reduced interfacial recombination losses. However, the performance of solution‐processed CuSbS_2_ HTLs is governed not only by their intrinsic electronic structure but also by film‐forming behavior, which is strongly dependent on the particle morphology arising from their growth mechanisms. A key limitation arises from the strong tendency of CuSbS_2_ to form micron‐sized anisotropic plate‐like structures when elemental sulfur (ES) is used as the anion precursor, resulting in non‐uniform and incomplete coverage on polycrystalline perovskite surfaces and the formation of interfacial voids that act as recombination centers, thereby rendering its unfavorable candidate for HTL applications [23, 24, 25]. The incorporation of smaller isotropic or quasi‐spherical CuSbS_2_ nanocrystals enables the filling of interstitial spaces between larger plate‐like domains, resulting in more compact packing, improved film continuity, and enhanced conformal interfacial and surface coverage. Therefore, suppressing excessive plate‐like overgrowth in CuSbS_2_ toward a mixed anisotropic/isotropic nanocrystal population is highly desirable for achieving high‐quality HTL layers. This morphological evolution is governed by the interplay between sulfur precursor chemistry and ligand coordination pathways. Sulfur sources exhibit distinct decomposition behaviors and sulfur‐release kinetics, which strongly influence growth anisotropy and defect formation during nanocrystal evolution [26, 27]. For instance, the hexamethyldisilathiane (TMS), as a rapidly releasing sulfur precursor, promotes fast nucleation and restricts lateral growth, resulting in reduced plate dimensions; on the other hand, thiourea (ThU) provides a slower sulfur supply that enables more controlled growth and supports the formation of smaller, mixed‐morphology nanocrystals [27]. In terms of ligand coordination, oleylamine (OAm) promotes anisotropic, plate‐like growth through selective facet binding, while oleic acid (OAc) introduces competitive coordination that suppresses directional growth, implying that the OAm/OAc ratio plays a key role in regulating facet stabilization and growth selectivity, offering an effective handle to tune particle morphology [28, 29]. Despite these factors, as far as we know, there is no comprehensive research on regulating the morphology and energetic disorder of CuSbS_2_ nanoparticles by controlling sulfur sources and ligand coordination in the precursor solution for their direct impact on HTL functionality in PSCs.

Herein, we report the size‐ and shape‐controlled synthesis of CuSbS_2_ nanocrystals via a hot‐injection approach by tuning the sulfur precursor (TMS and ThU) and ligand composition (OAm and OAc) to enable efficient and stable dopant‐free perovskite solar cells. Inspired by this perspective, reaction temperature, reaction time, and ligand ratio were optimized to elucidate their roles in directing crystal growth, and the impact of morphology in CuSbS_2_ HTLs on interfacial charge transfer and device performance was comprehensively evaluated in n–i–p architectures. The TMS‐based recipe retained plate‐like shapes with reduced lateral dimensions, whereas the thiourea precursor with an optimized OAm/OAc ratio (3:7) enabled simultaneous size reduction and the formation of mixed plate‐like/quasi‐spherical nanostructures. This mixed morphology not only promotes conformal coverage of the perovskite surface by filling voids between plate‐like domains but also suppresses non‐radiative recombination and energetic disorder. The obtained CuSbS_2_ layers were employed as hole transport layers in triple cation‐based PSCs and benchmarked against counterparts synthesized using elemental sulfur. The thiourea‐treated CuSbS_2_ device achieved a champion efficiency of 22.72% (*Voc* = 1.15 V, *Jsc* = 24.33 mA cm^−^^2^, *FF* = 0.82), outperforming both TMS‐treated CuSbS_2_ (20.00%) and ES‐treated CuSbS_2_ (17.31%). The optimized device also exhibited excellent environmental and operational stability, retaining ∼80% of its initial efficiency after 1000 h at 50–70% relative humidity, ∼80% after 30 days of thermal aging under N_2_, and ∼60% after 1000 h of continuous maximum power point tracking under LED illumination. These results demonstrate that sulfur‐source and ligand‐regulated morphology engineering is a critical design strategy for high‐performance inorganic HTLs in perovskite solar cells.

## 2 Results and Discussions

Figure 1 summarizes the precursor‐dependent growth concept and the resulting structural and morphological characteristics of CuSbS_2_ nanocrystals synthesized using hexamethyldisilathiane (TMS) and thiourea (ThU) as sulfur precursors, while the elemental sulfur‐derived reference sample (CSS‐ES) is presented in Figure S1. The CSS‐ES sample is included as a literature‐supported reference representing elemental sulfur‐mediated, plate‐dominated CuSbS_2_ growth, consistent with previous reports [30], and is therefore employed herein as the control material for evaluating sulfur‐precursor‐dependent nanocrystal growth and subsequent device behavior. For the TMS‐ and ThU‐assisted routes, a systematic stepwise optimization was carried out by sequentially varying the reaction temperature, reaction time, and OAm: OAc ratio, as summarized in Figures S2–S4 for CSS‐TMS and Figures S6–S8 for CSS‐ThU. In each case, the optimum condition identified at one stage was fixed and used in the subsequent optimization step. Based on the combined XRD and SEM analyses, the optimum synthesis conditions were determined to be 240°C, 5 min, and an OAm: OAc ratio of 3:7 for the TMS‐assisted route, and 260°C, 5 min, and an OAm: OAc ratio of 3:7 for the ThU‐assisted route. Precursor‐dependent morphology evolution can be rationalized within a LaMer‐type nucleation and growth mechanism, in which the sulfur precursor governs the rate of reactive sulfur generation and thereby directly controls the monomer concentration, supersaturation profile, nucleation burst, and subsequent crystal‐growth regime. As illustrated schematically in Figure 1a, the TMS precursor, owing to its higher reactivity and faster sulfur release, is expected to induce a sharp increase in monomer concentration, leading to rapid supersaturation and burst nucleation, followed by a growth‐dominated regime that favors anisotropic crystal extension along energetically preferred directions. This growth behavior promotes the formation of platelet‐dominated CuSbS_2_ nanocrystals [31, 32]. On the other side, thiourea undergoes a more gradual decomposition and sulfurization process, which moderates monomer supply and suppresses abrupt supersaturation spikes. Such a kinetically moderated pathway reduces excessive anisotropic overgrowth and enables a more balanced nucleation–growth mechanism, thereby allowing the coexistence of plate‐like nanocrystals with smaller quasi‐spherical particles. Besides, the OAm/OAc ligand environment also plays a critical role in regulating precursor conversion and crystal‐facet stabilization. OAm can coordinate with Cu and Sb precursor species, thereby modulating precursor reactivity and monomer generation kinetics, whereas OAc contributes to surface passivation and facet stabilization through carboxylate binding, helping to suppress excessive oriented attachment and uncontrolled coalescence. Accordingly, the optimized OAm: OAc ratio of 3:7 likely provides a favorable balance between precursor coordination and surface‐energy regulation, enabling sufficient crystallization while limiting overgrowth and aggregation.

**FIGURE 1:**
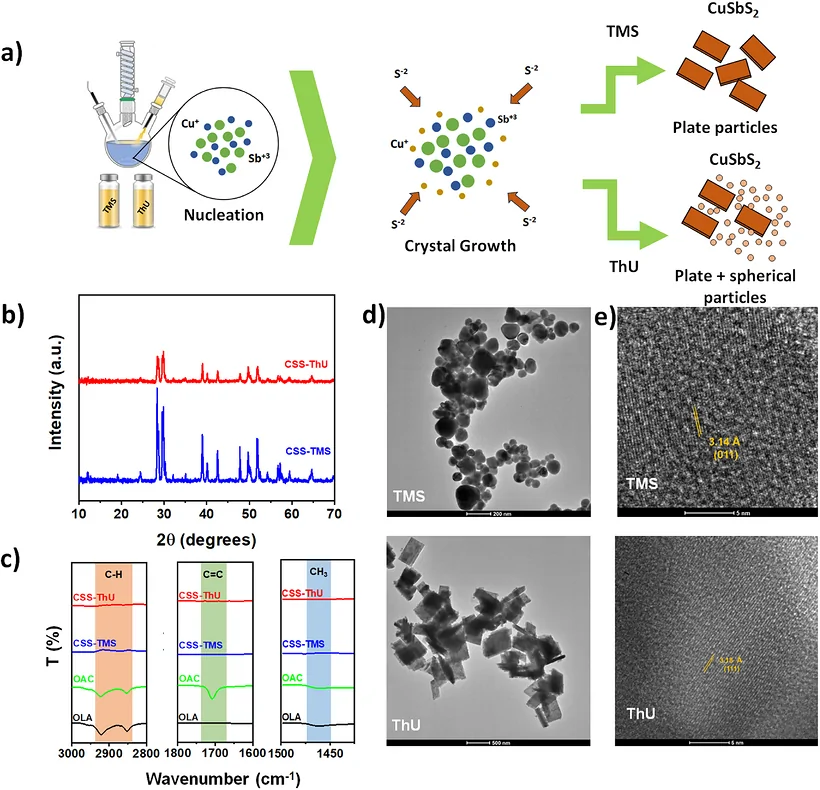
(a) Schematic illustration of the CuSbS_2_ nanoparticle formation pathway using different sulfur sources, highlighting the nucleation and crystal growth stages and the resulting morphology evolution from predominantly plate‐like particles in the TMS‐assisted route to a mixed plate/spherical particle distribution in the thiourea‐assisted route. (b) X‐ray diffraction (XRD) patterns of CuSbS_2_ nanoparticles synthesized using TMS (CSS‐TMS) and thiourea (CSS‐ThU). (c) Fourier‐transform infrared (FTIR) spectra of CuSbS_2_ nanoparticles and surface ligands/solvents, including OAc and OAm; (d) TEM images of CSS‐TMS and CSS‐ThU nanoparticles. (e) High‐resolution TEM (HR‐TEM) images of CSS‐TMS and CSS‐ThU, showing the lattice fringes of the synthesized CuSbS_2_ nanocrystals.

The crystal structures of optimized CSS‐TMS and CSS‐ThU were confirmed by X‐ray diffraction (Figure 1b). Both samples exhibit diffraction peaks that can be indexed to the orthorhombic CuSbS_2_ phase, confirming the successful formation of the target ternary chalcogenide structure [22]. No obvious impurity peaks are observed in the main diffraction patterns, indicating that both sulfur precursors can yield phase‐pure CuSbS_2_ under optimized conditions. Nevertheless, minor differences in relative peak intensities suggest that the sulfur precursor affects the preferred growth orientation and crystallographic texture, in agreement with the distinct morphology evolution observed by electron microscopy [31, 33]. The stepwise phase formation and morphology optimization are further supported by the temperature‐, time‐, and OAm: OAc‐ratio‐dependent XRD/SEM studies shown in Figures S2–S4 for the TMS route and Figures S6–S8 for the ThU route, while the structural validity of the elemental sulfur‐derived reference sample is confirmed in Figure S1. Fourier‐transform infrared spectroscopy (FTIR) in Figure 1c further reveals the characteristic vibrational features associated with the OAm and OAc ligands. Compared with the spectra before purification (Figure S5), the ligand‐related absorption bands become substantially weaker after the washing process, indicating that most surface‐bound organic ligands are effectively removed and that any remaining ligand species are probably present at negligible levels. More importantly, no additional bands attributable to precursor‐derived organic residues are observed, indicating effective precursor conversion for both sulfur‐precursor routes. The optimized ligand coordination environment and crystallographic consistency are further supported by the comparative FTIR and SAED analyses provided in Figures S5 and S9. The most pronounced precursor‐dependent difference is revealed by transmission electron microscopy (TEM) in Figure 1d. The CSS‐TMS sample is dominated by large anisotropic plate‐like nanocrystals, indicating that the rapid sulfur release from TMS promotes directional growth and the formation of extended platelet domains. Although such a morphology reflects good crystallinity, it is less favorable for interfacial thin‐film formation because large platelets tend to stack inefficiently and can generate interparticle voids on rough perovskite surfaces. In contrast, the CSS‐ThU sample exhibits a distinctly different morphology composed of plate‐like nanocrystals together with a substantial population of quasi‐spherical nanoparticles. This mixed architecture is highly advantageous because the smaller quasi‐spherical particles can fill the gaps between larger plate‐like domains, thereby improving packing density, reducing void formation, and promoting a more continuous HTL layer. High‐resolution TEM (HR‐TEM) in Figure 1e further confirms the crystalline nature of both samples. Well‐resolved lattice fringes are observed for both CSS‐TMS and CSS‐ThU, and the measured interplanar spacings are consistent with the characteristic lattice planes of orthorhombic CuSbS_2_, in agreement with the XRD results [33]. Importantly, the CSS‐ThU sample maintains clear lattice ordering despite its more complex mixed morphology, indicating that suppressing excessive anisotropic overgrowth does not compromise crystallinity. These results demonstrate that while all three sulfur sources can produce crystalline CuSbS_2_ nanocrystals, sulfur precursor chemistry critically determines the nucleation–growth balance, growth anisotropy, and final particle architecture, with the thiourea route providing the most favorable morphology for constructing dense and conformal inorganic HTLs.

To further elucidate the sulfur‐precursor‐dependent differences in the chemical and electronic properties of CuSbS_2_, the optimized CSS‐ES, CSS‐TMS, and CSS‐ThU samples were systematically investigated by XPS, diffuse reflectance spectroscopy, UPS, and conductivity measurements. The optimized CSS‐TMS and CSS‐ThU samples were then comparatively analyzed in detail in Figure 2. The high‐resolution XPS spectra of Cu, Sb, and S (Figure 2a–c) confirm the successful formation of CuSbS_2_ in both samples. As shown in Figure 2a, the characteristic Cu 2p_3_/_2_ and Cu 2p_1_/_2_ peaks are located at approximately 932.5 and 952.3 eV, respectively, indicating that copper is predominantly present in the Cu^+^ oxidation state. The absence of pronounced satellite features further confirms the negligible presence of Cu^2^^+^ species. Compared with CSS‐TMS, the CSS‐ThU sample exhibits slightly sharper and more symmetric Cu 2p peaks. The Sb 3d spectra (Figure 2b) display the characteristic Sb 3d_5_/_2_ and Sb 3d_3_/_2_ peaks at approximately 529–530 and 538–539 eV, respectively, which are consistent with Sb^3^^+^ in the CuSbS_2_ lattice [34, 35]. These results further verify the successful formation of the target ternary sulfide structure in both samples. Compared with CSS‐TMS, the Sb 3d peaks of CSS‐ThU also exhibit a more regular spectral profile. A more pronounced difference is observed in the S 2p region (Figure 2c). In both samples, a broad spectral feature appears in the 161–163 eV range, corresponding to the overlapping S 2p_3_/_2_ and S 2p_1_/_2_ components, confirming that sulfur predominantly exists in the S^2^^−^ state within the metal sulfide framework [36]. Compared with CSS‐TMS, the CSS‐ThU sample exhibits a more compact and better‐defined S 2p profile. The consistently sharper and more regular Cu 2p, Sb 3d, and S 2p peak profiles observed for the CSS‐ThU sample indicate a narrower distribution of local chemical environments within the CuSbS_2_ lattice. This behavior can be attributed to the slower sulfur‐release kinetics of thiourea, which promote a more balanced precursor conversion process and more controlled sulfur incorporation during crystal growth [31, 35, 37]. As a result, chemically non‐uniform regions and sulfur‐deficient local environments are effectively suppressed, leading to a more homogeneous Cu─Sb─S coordination network with fewer defect‐prone surface states. On the other hand, the faster sulfur release associated with the TMS‐assisted route likely results in less regulated sulfurization and greater local compositional heterogeneity. Such differences are particularly important for HTL applications, where sulfur‐vacancy‐related trap states can intensify interfacial recombination and hinder selective hole extraction. The UPS results (Figure 2d,e) reveal that both CSS‐TMS and CSS‐ThU possess band‐edge positions suitable for hole extraction from the triple‐cation perovskite absorber. The optical band gaps estimated from the diffuse reflectance spectra using the Kubelka–Munk method (Figure S10a,b) are approximately 1.52 eV for both samples, while the corresponding baseline optical response of CSS‐ES is provided in Figure S1f. As summarized in the energy‐level diagram (Figure 2f), the valence‐band maximum (VBM) values of CSS‐TMS and CSS‐ThU are approximately −5.41 and −5.42 eV, respectively, with corresponding conduction‐band minimum (CBM) values of approximately −3.89 and −3.90 eV. The minor difference of approximately 0.01 eV does not indicate a substantial sulfur‐precursor‐induced shift in the intrinsic band‐edge positions, but instead confirms that both CuSbS_2_ HTLs possess comparably suitable energetic alignment for selective hole extraction and electron blocking. The principal electronic distinction between the samples is more clearly reflected in their current–voltage characteristics (Figure S10c). CSS‐ThU exhibits the steepest current–voltage slope among the three CuSbS_2_ samples, indicating the highest electrical conductivity, whereas CSS‐ES shows the lowest and CSS‐TMS an intermediate response. The enhanced conductivity of CSS‐ThU is consistent with its more homogeneous local chemical environment and more effective interparticle connectivity arising from the mixed plate‐like/quasi‐spherical morphology. On the other hand, the larger platelet‐dominated domains of CSS‐TMS and the more anisotropic morphology of CSS‐ES are expected to limit particle packing and charge percolation within the HTL film. To further quantify the energetic disorder of the CuSbS_2_ HTLs, temperature‐dependent hole mobility measurements were performed between 180 and 340 K and analyzed using the Gaussian disorder model (Figure S11). The extracted energetic disorder coefficients (σ) are 75 meV for CSS‐ES, 54 meV for CSS‐TMS, and 35 meV for CSS‐ThU, demonstrating a progressive reduction in energetic disorder with increasing control over sulfur‐release kinetics. The remarkably lower σ value of CSS‐ThU indicates a narrower distribution of localized electronic states, which is consistent with its improved chemical homogeneity and enhanced charge‐transport characteristics. These results demonstrate that the thiourea‐assisted synthesis enables the formation of a chemically cleaner, electrically more conductive, energetically well‐matched, and lower‐disorder CuSbS_2_ HTL for efficient hole transport.

**FIGURE 2:**
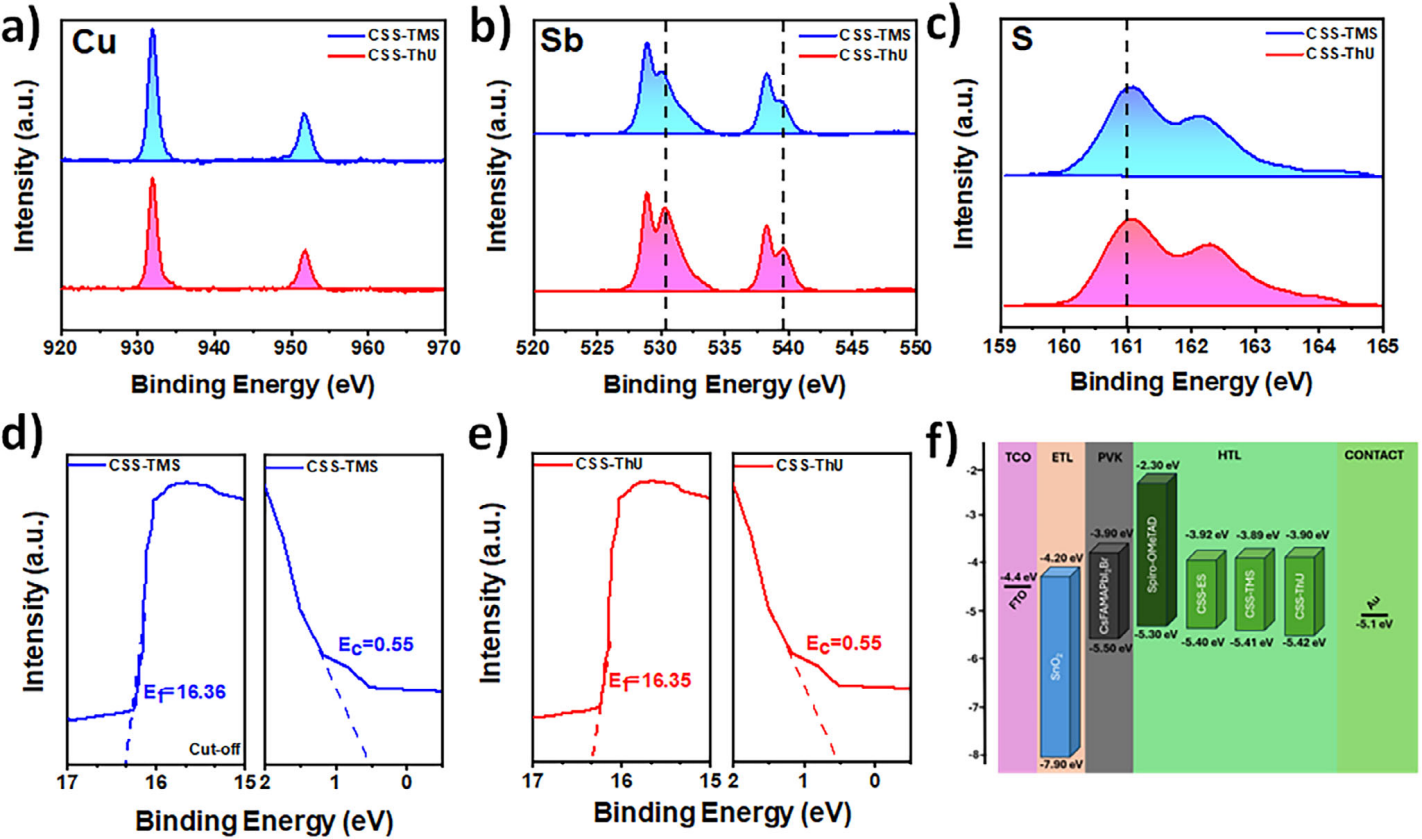
High‐resolution XPS spectra of (a) Cu, (b) Sb, and (c) S for optimized CuSbS_2_ nanoparticles synthesized using TMS (CSS‐TMS) and thiourea (CSS‐ThU), highlighting the sulfur‐precursor‐dependent surface chemical states. UPS spectra of (d) CSS‐TMS and (e) CSS‐ThU used to determine the valence‐band edge and electronic structure. (f) Schematic energy‐level alignment of the device stack, illustrating the band positions of the CuSbS_2_ hole‐transport layers relative to the triple‐cation absorber and adjacent layers.

After structural, chemical, and electronic optimization of the CuSbS_2_ nanocrystals, their applicability as inorganic hole‐transport layers was evaluated in n–i–p perovskite solar cells with the FTO/SnO_2_/Cs_0.05_FA_0.85_MA_0.1_Pb(I_0.85_Br_0.15_)_3_/HTL/Au architecture, and the corresponding device performance is summarized in Figure 3. As illustrated in Figure 3a, the distinct particle morphologies of CSS‐ES, CSS‐TMS, and CSS‐ThU lead to markedly different interfacial layer characteristics on the perovskite surface. The CSS‐ES film, dominated by anisotropic plate‐like particles, forms a comparatively less compact top layer with larger interparticle gaps, while the CSS‐TMS sample shows a partially improved but still predominantly platelet‐like morphology. On the other hand, the mixed plate‐like/quasi‐spherical morphology of CSS‐ThU enables a denser and more conformal interfacial coating. Cross‐sectional SEM images further support that the thiourea‐derived CuSbS_2_ establishes a more continuous HTL/perovskite interface, which is expected to improve interfacial coverage, enhance charge selectivity, and suppress direct recombination pathways at the back contact. To quantitatively compare the surface topography of the CuSbS_2_‐coated perovskite films, AFM measurements were performed after deposition of the CSS‐ES, CSS‐TMS, and CSS‐ThU hole‐transport layers (Figure S12). The root‐mean‐square surface roughness decreases systematically from 17.05 nm for CSS‐ES to 10.52 nm for CSS‐TMS and 8.57 nm for CSS‐ThU. The significantly lower roughness of the CSS‐ThU‐coated film demonstrates that the thiourea‐derived nanocrystals produce a smoother and more homogeneous surface than others. These findings are consistent with the cross‐sectional SEM observations and further support that the mixed nanocrystal morphology of CSS‐ThU promotes the formation of a more uniform HTL surface on the perovskite layer. The photovoltaic response is first reflected in the *J–V* curves shown in Figure 3b. Among the three device configurations, the CSS‐ThU‐based perovskite solar cells exhibit the highest overall photovoltaic output with a smaller discrepancy between forward and reverse scans, indicating reduced hysteretic behavior. Notably, the CSS‐ThU device achieved a *Jsc* of 24.33 mA cm^−^^2^, *Voc* of 1.15 V, *FF* of 0.82, and a PCE of 22.72%, outperforming both CSS‐TMS (22.85 mA cm^−^^2^, 1.10 V, 0.79, 20.00%) and CSS‐ES (21.90 mA cm^−^^2^, 1.04 V, 0.76, 17.31%), respectively. Detailed photovoltaic parameters are summarized in Table 1. These results clearly demonstrate that sulfur‐precursor engineering of CuSbS_2_ directly translates into progressive device‐level improvements, establishing a clear performance hierarchy of CSS‐ES < CSS‐TMS < CSS‐ThU. To further benchmark the performance of our devices, Table S1 summarizes previously reported perovskite solar cells employing ternary chalcogenide‐based inorganic HTLs. Although the photovoltaic metrics remain below those of the highest‐performing organic HTM‐based PSCs, the efficiency and stability achieved in this work are highly competitive within the class of inorganic chalcogenide HTLs and provide important design insights for future inorganic HTL engineering. The superiority of the thiourea‐derived HTL is further supported by the EQE spectra shown in Figure 3c. All devices exhibit broad photo‐response across the visible region, confirming efficient light harvesting by the triple cation absorber. As expected, the CSS‐ThU‐based device consistently shows the highest EQE intensity over a wide wavelength range, resulting in the largest integrated current density. This behavior indicates more efficient carrier collection, which is consistent with its more favorable morphology, higher conductivity, and improved interfacial energy‐level alignment [17]. Meanwhile, the lower EQE response of the CSS‐ES device and the intermediate response of CSS‐TMS suggest less efficient charge extraction and/or increased recombination losses, in agreement with their less compact interfacial coverage. The device‐to‐device reproducibility shown in the PCE box plots (Figure 3d) further confirms that the performance enhancement is not limited to isolated high‐performing devices. Based on 30 independent devices, the CSS‐ThU group exhibits the highest median PCE with a narrower distribution, indicating both improved efficiency and better reproducibility. On the other side, the CSS‐ES devices show the lowest efficiency, while CSS‐TMS displays intermediate performance, consistent with the gradual improvement in HTL quality and interfacial contact. This interpretation is further supported by the FF‐loss analysis in Figure 3e, where the CSS‐ThU‐based devices display reduced fill‐factor loss compared with both CSS‐ES and CSS‐TMS, indicating more efficient charge transport and suppressed non‐radiative recombination [38]. In particular, the CSS‐ES device exhibits the largest charge‐transport‐related FF loss, while CSS‐TMS shows a reduced loss and CSS‐ThU approaches the lowest transport dynamics, consistent with progressively improved interparticle connectivity and interfacial charge extraction. Considering both the optimized deposition conditions (Figure S13) and the superior steady‐state power output under continuous illumination (Figure S14), these results demonstrate that thiourea‐assisted CuSbS_2_ functions as a highly effective inorganic HTL, delivering higher efficiency, reduced hysteresis, improved reproducibility, and superior operational performance in triple cation‐based perovskite solar cells.

**FIGURE 3:**
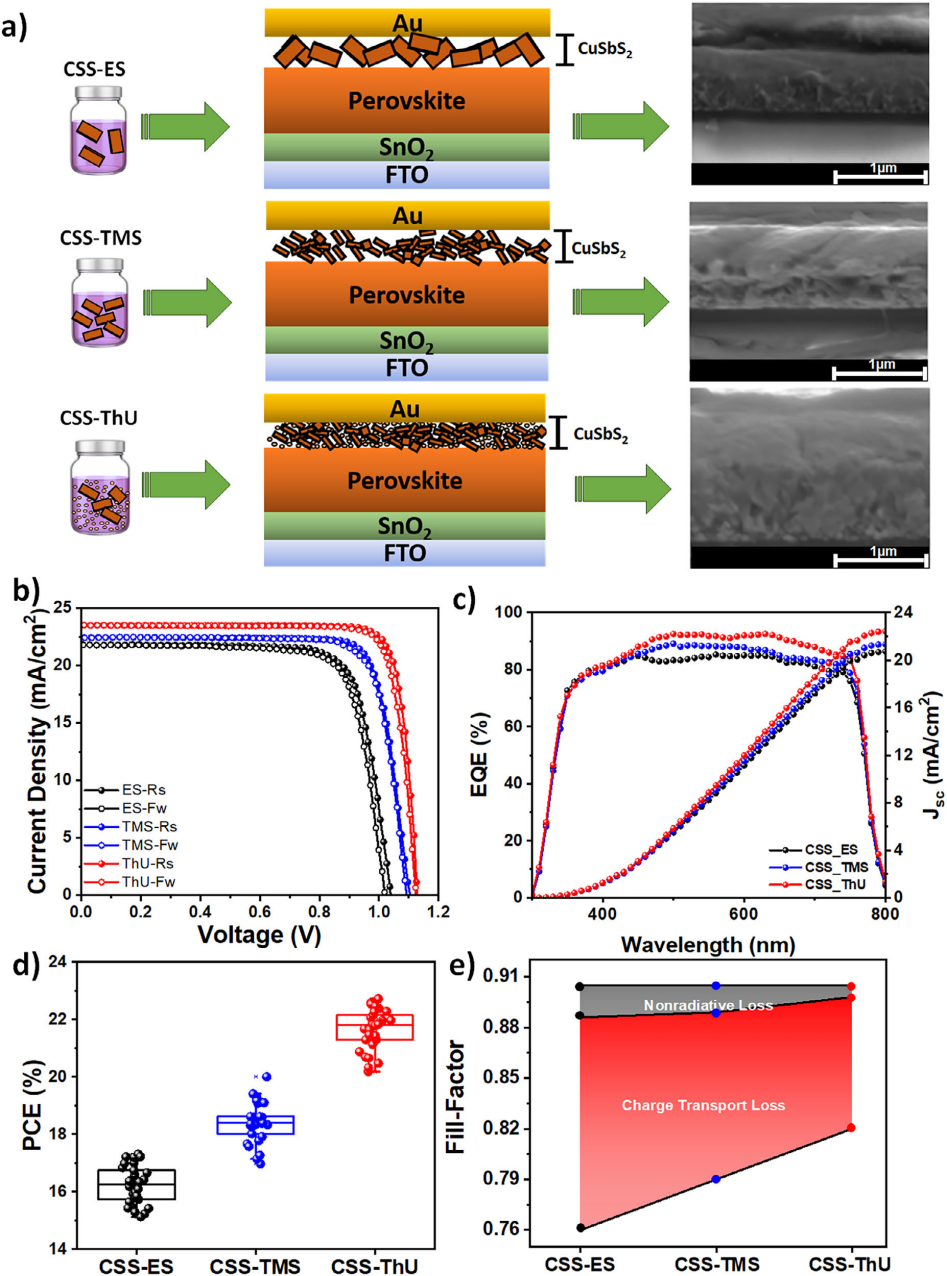
(a) Schematic illustration of the integration of CSS‐TMS and CSS‐ThU CuSbS_2_ nanocrystals as hole‐transport layers (HTLs) on top of the triple cation perovskite absorber, together with representative cross‐sectional SEM images highlighting the morphology‐dependent HTL/perovskite interface. (b) External quantum efficiency (EQE) spectra and integrated current densities (*Jsc*) of perovskite solar cells employing CSS‐ES, CSS‐TMS, and CSS‐ThU as HTLs. (c) Forward and reverse *J–V* curves of the corresponding devices. (d) Statistical box plots of power conversion efficiency (PCE) obtained from 30 independent devices for each HTL. (e) Fill‐factor‐loss (FF‐loss) analysis, comparing charge‐transport‐related and non‐radiative‐loss contributions among the control, CSS‐TMS, and CSS‐ThU devices.

**TABLE 1:** Photovoltaic performance parameters of perovskite solar cells employing different hole‐transport layers, including CSS‐TMS, CSS‐ThU, and CSS‐ES.

HTL Type		*Jsc* (mA/cm^2^)	*Voc* (V)	*FF*	PCE (%)
**CSS‐ES**	Average	20.76±0.783	1.04±0.010	0.75±0.006	16.23±0.641
	Champion	21.9	1.04	0.76	17.31
**CSS‐TMS**	Average	21.39±0.68	1.1±0.008	0.78±0.005	18.4±0.674
	Champion	22.85	1.1	0.79	20
**CSS‐ThU**	Average	23.33±0.71	1.13±0.009	0.82±0.007	21.65±0.683
	Champion	24.33	1.15	0.82	22.72

To directly probe the chemical interaction at the CuSbS_2_/perovskite interface, depth‐dependent XPS measurements were performed on a fractured cross‐section of the device by collecting spectra from three representative regions, namely the upper CuSbS_2_ surface, the CuSbS_2_/perovskite interface, and the bottom perovskite region (Figure S15). As expected, only sulfur signals are detected on the upper CuSbS_2_ surface, whereas Pb 4f and I 3d peaks emerge together with the S 2p signal at the interface, confirming the formation of the CuSbS_2_/perovskite heterojunction. Meanwhile, the bottom perovskite region is dominated by Pb and I signals, while no detectable sulfur signal is observed, indicating that sulfur remains confined to the CuSbS_2_ layer with negligible diffusion into the underlying perovskite. More importantly, the high‐resolution spectra reveal distinct binding‐energy shifts across the heterointerface. Both the Pb 4f and S 2p peaks shift toward lower binding energies, whereas the I 3d peak exhibits a clear shift toward higher binding energy. These opposite spectral shifts provide direct evidence of interfacial charge redistribution induced by electronic coupling between the sulfur‐rich CuSbS_2_ surface and undercoordinated Pb species at the perovskite surface. The lower binding energies of Pb 4f and S 2p indicate an altered local electronic environment arising from sulfur‐mediated coordination at the heterointerface, which partially redistributes electron density and modifies the electrostatic screening of both atomic species. On the other hand, the positive shift of the I 3d peak is consistent with a decrease in electron density around iodide ions owing to the reorganization of the local Pb─I coordination environment after sulfur‐mediated interfacial passivation. Such electronic redistribution is expected to reduce the population of undercoordinated interfacial defect sites and suppress trap‐assisted recombination, thereby establishing a chemically and electronically more favorable interface for hole extraction.

To further elucidate the impact of the interfacial electronic characteristics on carrier transport and recombination, complementary electrical and spectroscopic measurements were carried out, and the corresponding results are summarized in Figure 4. The Mott–Schottky plots (Figure 4a) reveal that the built‐in potential increases progressively from 0.90 V for CSS‐ES to 0.96 V for CSS‐TMS and reaches the highest value of 1.05 V for CSS‐ThU, indicating that the thiourea‐derived CuSbS_2_ establishes the most favorable internal electric field for charge separation and carrier extraction [39]. This result is consistent with the improved band alignment and more conformal HTL morphology observed in the above‐mentioned findings. The reduction in defect‐assisted losses is further supported by the space‐charge‐limited current (SCLC) analysis (Figure 4b). The trap‐filled limit voltages (V_TFL_) were extracted as 0.26 V for CSS‐ES, 0.24 V for CSS‐TMS, and 0.22 V for CSS‐ThU. Notably, the CSS‐ThU device exhibits the lowest V_TFL_, implying the lowest trap‐state density among the compared CuSbS_2_‐based hole‐transport layers. This result points to a more defect‐tolerant charge‐transport pathway and a reduced density of interfacial trap states, consistent with the formation of a cleaner and more electronically selective HTL/perovskite interface in the thiourea‐derived system. To further clarify the recombination dynamics, the devices were subsequently analyzed through the Voc–light intensity dependence and electrochemical impedance spectroscopy (EIS). As shown in Figure 4c, the slope decreases from 1.59 kT/q for CSS‐ES to 1.24 kT/q for CSS‐TMS and further to 1.15 kT/q for CSS‐ThU, indicating that trap‐assisted Shockley–Read–Hall recombination is most effectively suppressed in the thiourea‐derived device [40, 41]. This interpretation is supported by the Nyquist plots in Figure 4d, where CSS‐ThU exhibits the largest semicircle radius, corresponding to the highest recombination resistance among the three devices [42]. Moreover, these results confirm that the CSS‐ThU‐based device possesses the most recombination‐resistant and electronically selective HTL/perovskite interface. The steady‐state PL and TRPL measurements (Figure 4e,f) provide further insight into the interfacial carrier dynamics. Compared with CSS‐ES and CSS‐TMS, the CSS‐ThU device shows the strongest PL quenching, indicating more efficient interfacial hole extraction from the perovskite absorber [39, 40]. At the same time, the TRPL decay of the CSS‐ThU sample exhibits the longest effective carrier lifetime (τ ≈ 25 ns), compared with CSS‐TMS (τ ≈ 22 ns) and CSS‐ES (τ ≈ 21 ns). Rather than indicating slower extraction, this behavior suggests that the thiourea‐derived CuSbS_2_ more effectively suppresses non‐radiative recombination and trap‐assisted carrier loss, allowing photogenerated carriers to survive longer while still being extracted more selectively at the interface. These results demonstrate that the thiourea‐derived CuSbS_2_ HTL exhibits the highest built‐in potential, minimized trap‐assisted and non‐radiative recombination losses, and the most efficient interfacial charge extraction, thereby providing the mechanistic basis for the superior efficiency and operational stability of the CSS‐ThU‐based perovskite solar cells.

**FIGURE 4:**
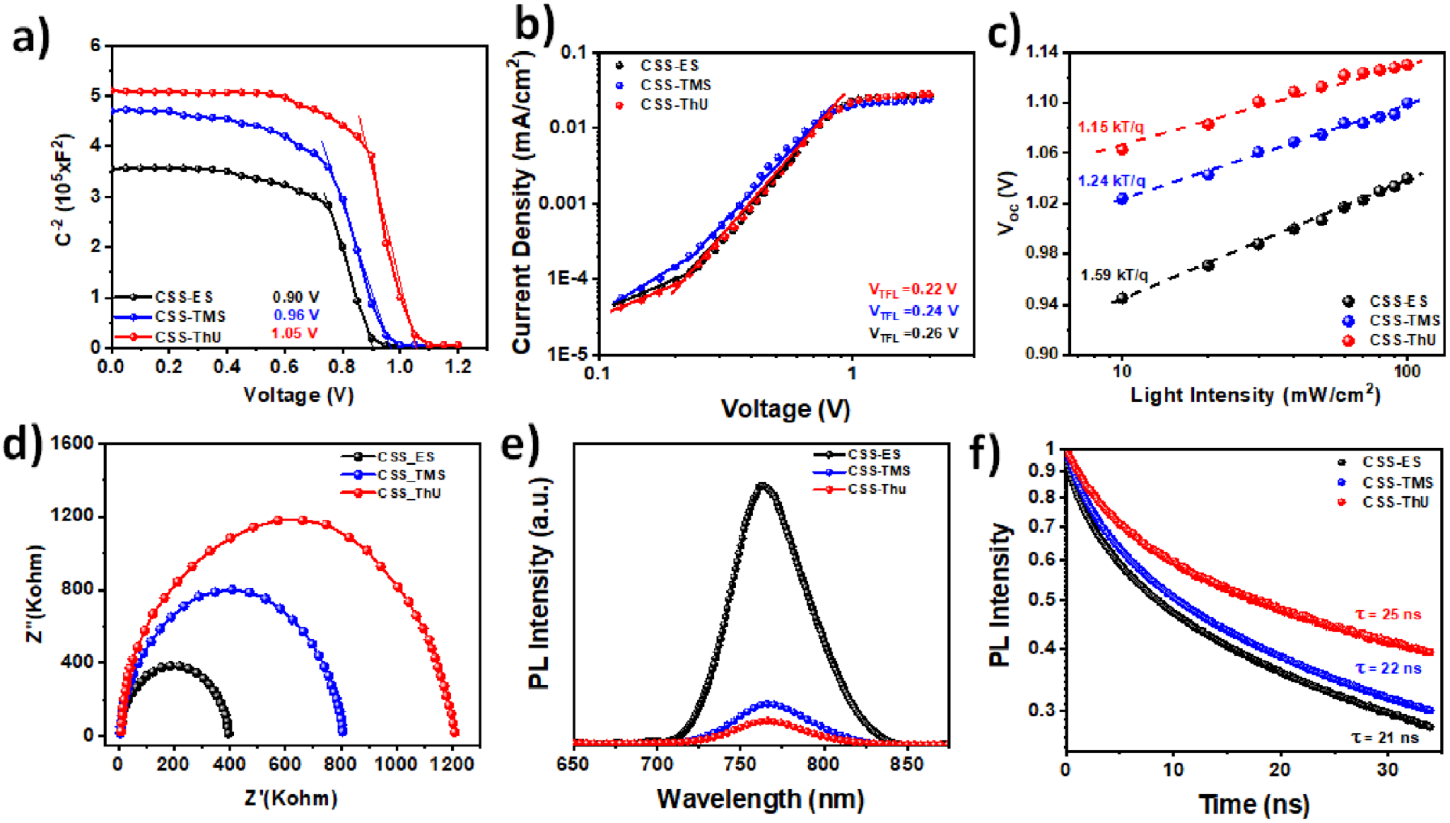
Charge‐transport and recombination analyses of perovskite solar cells employing CSS‐ES, CSS‐TMS, and CSS‐ThU as hole‐transport layers. (a) Mott–Schottky plots extracted from capacitance–voltage measurements. (b) Space‐charge‐limited current (SCLC) characteristics used to evaluate the trap‐filled limit voltage (V_TFL_) and trap‐related transport behavior. (c) Dependence of open‐circuit voltage (Voc) on light intensity. (d) Nyquist plots obtained from electrochemical impedance spectroscopy (EIS). (e) Steady‐state photoluminescence (PL) spectra. (f) Time‐resolved photoluminescence (TRPL) decay curves.

Figure 5 summarizes the intrinsic stability, structural durability, surface wettability, and long‐term shelf, thermal with operational stability of PSCs employing CSS‐ES, CSS‐TMS, and CSS‐ThU as CuSbS_2_‐based hole‐transport layers, highlighting the remarkable stability enhancement achieved through thiourea‐assisted sulfur precursor engineering. The intrinsic stability of the CuSbS_2_ nanocrystals was first evaluated by monitoring the evolution of their optical absorption during ambient aging. As shown in Figure 5a, all samples exhibit a gradual decrease in absorbance with increasing aging time up to 240 h, although the degradation rate strongly depends on the sulfur precursor employed during synthesis. Among the three samples, CSS‐ThU maintains nearly unchanged absorbance throughout the visible region, particularly beyond ∼550 nm, even after 240 h of aging, whereas CSS‐ES undergoes the most pronounced attenuation over the entire spectral range. CSS‐TMS exhibits intermediate behavior, with a noticeable reduction in visible‐light absorption after approximately 120 h. These results indicate that the thiourea‐derived CuSbS_2_ nanocrystals possess substantially improved resistance to environmental degradation, reflecting their enhanced chemical stability and structural robustness. The structural stability of the CuSbS_2_‐based PSCs was further evaluated by X‐ray diffraction after prolonged aging (Figure 5b). Although the characteristic diffraction peaks of the perovskite absorber remain largely preserved for all devices, clear differences are observed in the formation of the degradation product. In particular, the PbI_2_ diffraction peak (marked by asterisks) becomes most pronounced for the CSS‐ES device, less apparent for CSS‐TMS, and only weakly detectable for CSS‐ThU. The significantly suppressed PbI_2_ formation demonstrates that the thiourea‐derived CuSbS_2_ HTL more effectively preserves the structural integrity of the perovskite during aging. These observations indicate that sulfur‐precursor engineering significantly mitigates structural degradation of the perovskite absorber. As shown in Figure S16, the static water contact angle increases from 87.74° for CSS‐ES to 89.55° for CSS‐TMS and further to 102.30° for CSS‐ThU, demonstrating that the thiourea‐derived CuSbS_2_ layer possesses the most hydrophobic surface among the investigated HTLs. The increased hydrophobicity is expected to suppress moisture adsorption and retard water penetration toward the buried perovskite/HTL interface [41]. Moreover, the improved film uniformity and compact nanoparticle packing of the CSS‐ThU layer are expected to minimize interparticle voids and provide more conformal interfacial coverage, thereby reducing the exposure of chemically vulnerable regions to environmental stress. The practical impacts of these material characteristics are directly reflected in the stability tests. During humidity aging at 25°C under 50–70% relative humidity in the dark for 1000 h (Figure 5c), the CSS‐ES device exhibits severe degradation, retaining only approximately 12% of its initial PCE. In comparison, the CSS‐TMS and CSS‐ThU devices retain approximately 65% and 78% of their initial efficiencies, respectively. This superior moisture tolerance of CSS‐ThU is consistent with its higher surface hydrophobicity and denser interfacial coverage, both of which can inhibit moisture ingress and delay moisture‐triggered degradation processes. Thermal stability measurements performed in the dark under N_2_ atmosphere for 30 days (0–10 days at 65°C, followed by 10–30 days at 85°C) in Figure 5d further confirm the robustness of the CuSbS_2_‐based HTLs, with CSS‐ThU again showing the best performance by retaining approximately ∼78–80% of its initial efficiency after the full aging period, while CSS‐TMS and CSS‐ES retain only ∼63% and ∼37%, respectively. This improved thermal stability can be attributed to the inorganic and dopant‐free nature of the CuSbS_2_ HTL, together with the more uniform and mechanically stable interfacial contact provided by the thiourea‐derived layer. The lower thermally induced degradation of CSS‐ThU suggests that the thiourea‐derived HTL more effectively suppresses interfacial decomposition, charge accumulation, and morphology‐induced local failure under prolonged thermal stress [43]. The operational stability of the devices was further evaluated under continuous maximum power point tracking under 100 mW cm^−^^2^ LED illumination in a nitrogen atmosphere at 65°C for 1000 h (Figure 5e). Under these conditions, the CSS‐ES device retains approximately 25% of its initial PCE, whereas the CSS‐TMS and CSS‐ThU devices retain approximately 41% and 58%, respectively,, confirming the improved operational durability of the thiourea‐derived CuSbS_2_ HTL under prolonged illumination and thermal stress. These results demonstrate that CSS‐ThU delivers markedly superior stability compared with both CSS‐ES and CSS‐TMS because its more hydrophobic and compact interfacial architecture more effectively suppresses moisture ingress, charge accumulation, and thermally activated degradation, highlighting the importance of both sulfur‐precursor and ligand engineering on CuSbS_2_ for durable perovskite solar cells.

**FIGURE 5:**
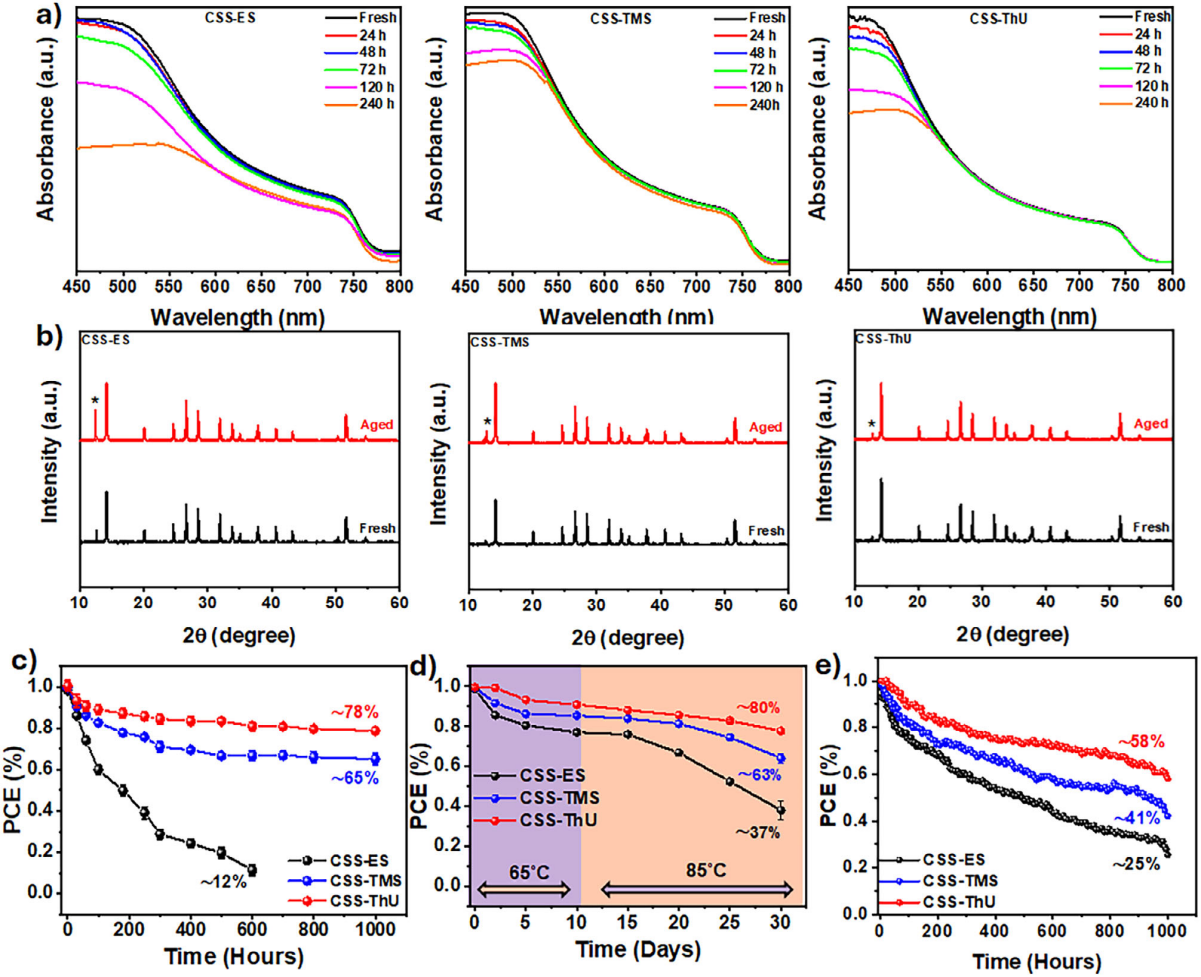
(a) UV–Vis absorption spectra of perovskite film deposited by CSS‐ES, CSS‐TMS, and CSS‐ThU nanocrystals recorded after different ambient aging times (0–240 h). (b) XRD patterns of fresh and aged perovskite solar cells employing CSS‐ES, CSS‐TMS, and CSS‐ThU hole‐transport layers after 10 days of aging. The diffraction peak of PbI_2_ is marked by asterisks. (c) Humidity stability of unencapsulated perovskite solar cells stored at 25°C under 50–70% relative humidity in the dark for 1000 h. (d) Thermal stability of unencapsulated devices measured in a nitrogen atmosphere for 30 days (0–10 days at 65°C, followed by 10–30 days at 85°C). (e) Operational stability of unencapsulated devices under continuous maximum power point tracking (MPPT) under 100 mW cm^−^^2^ LED illumination in a nitrogen atmosphere at 65°C for 1000 h.

## 3 Conclusion

We demonstrate that sulfur‐precursor and ligand engineering provides an effective strategy to regulate the nucleation, growth, and interfacial organization of CuSbS_2_ nanocrystals for dopant‐free inorganic hole‐transport layers in triple cation‐based n–i–p perovskite solar cells. By introducing elemental sulfur‐derived CuSbS_2_ (CSS‐ES) as a control group and systematically comparing it with TMS‐ and thiourea‐derived counterparts, we show that the sulfur‐release pathway critically determines nanocrystal morphology, surface chemistry, electronic structure, and interfacial functionality. In particular, the thiourea‐assisted route effectively suppresses excessive anisotropic platelet overgrowth and promotes a mixed plate‐like/quasi‐spherical CuSbS_2_ nanostructure that enables denser particle packing, more conformal interfacial coverage, and improved charge‐transport dynamics. As a result, the optimized CSS‐ThU device achieves the best photovoltaic performance, delivering a champion PCE of 22.72%, compared with 20.00% for CSS‐TMS and 17.31% for CSS‐ES. More importantly, the CSS‐ThU‐based devices exhibit significantly improved stability, retaining ∼78% of their initial efficiency after 1000 h under humid conditions, ∼78–80% after 30 days of thermal aging, and ∼58% after 1000 h under ISOS‐L2I conditions. This work provides a practical and scalable strategy for advancing CuSbS_2_‐based inorganic HTLs, where device performance is governed not only by the intrinsic electronic properties of chalcogenides but also by sulfur‐precursor‐directed control of chemical homogeneity, morphology, and interfacial organization, thereby enabling more efficient hole transport and stable next‐generation perovskite photovoltaics.

## Author Contributions

**Savas Sonmezoglu**: conceptualization, funding acquisition, Writing – review and editing, methodology, investigation, project administration, supervision, resources. **Ibrahimhan Dilci**: methodology, writing – original draft, formal analysis, visualization.

## Conflicts of Interest

The authors declare no conflicts of interest.

## Supporting information




**Supporting File 1**: advs76977‐sup‐0001‐SuppMat.docx.

## Data Availability

The data that support the findings of this study are available from the corresponding author upon reasonable request.
