# Supplementary material for: Suppressing Morphological and Energetic Disorder in Copper Antimony Sulfide‐based Hole‐Transporting Materials via Ligand–Precursor Engineering for Efficient and Stable Perovskite Solar Cells
Source: Adv Sci (Weinh). 2026 Jul 31:e76977. Online ahead of print. doi: 10.1002/advs.76977 (PMC13427232; doi:10.1002/advs.76977)
Supplement: Supplementary file 1 — Supporting File 1: advs76977‐sup‐0001‐SuppMat.docx. [file ADVS-9999-e76977-s001.docx]

**SUPPORTING INFORMATION**

**Suppressing Morphological and Energetic Disorder in Copper Antimony Sulfide-based Hole-Transporting Materials via Ligand–Precursor Engineering for Efficient and Stable Perovskite Solar Cells**

Ibrahimhan Dilci^a,b^, Savas Sonmezoglu^a,b,*^

^a^ Department of Metallurgical and Materials Engineering, Karamanoglu Mehmetbey University, 70100, Karaman, Türkiye

^b^ Nanotechnology R&D Laboratory, Karamanoglu Mehmetbey University, 70100, Karaman, Türkiye

^*^ Authors for correspondence:

Email address: ^1^[svssonmezoglu@kmu.edu.tr](mailto:svssonmezoglu@kmu.edu.tr) (S.SONMEZOGLU)

**Materials**

*N,N*-Dimethylformamide (DMF) and dimethyl sulfoxide (DMSO) were purchased from TCI. Cesium iodide (CsI, 99.999%), lead bromide (PbBr_2_​, ≥98%), lead iodide (PbI_2_​), methylammonium iodide (MAI), formamidinium iodide (FAI), hexamethyldisilathiane (TMS, synthesis grade), elemental sulfur, thiourea were obtained from Sigma-Aldrich. Antimony(III) chloride (SbCl_3_​) was supplied by Merck. Oleylamine (approximately 80–90% C_18_ content) was purchased from Acros Organics, while oleic acid (technical grade, 90%) and copper(I) chloride (CuCl, 97%) were obtained from Alfa Aesar.

**Synthesis of CuSbS_2_ Nanocrystals**

CuSbS_2_​ nanocrystals were synthesized via a hot-injection method under an inert nitrogen atmosphere. The synthesis setup consisted of a three-neck round-bottom flask equipped with a heating mantle, glass valves for gas inlet and outlet, a thermocouple, and an external cooling fan to facilitate post-reaction cooling. High-purity nitrogen gas was continuously supplied throughout the synthesis process. For the synthesis, CuCl (0.45 mmol) and SbCl_3_​ (0.45 mmol) were dissolved in 10 mL of oleylamine (OAm) at 100 °C under nitrogen for 1 h, corresponding to a Cu:Sb molar ratio of 1:1. The sulfur precursor amount was adjusted to achieve a Cu:Sb:S molar ratio of 1:1:2.5. Hexamethyldisilathiane (TMS) and thiourea (ThU) were employed as sulfur sources for the preparation of CuSbS_2_​ nanocrystals. After complete dissolution of the metal precursors, the reaction temperature was gradually increased to the desired injection temperature. The injection temperature was systematically optimized in the ranges of 180–280 °C for TMS and 200–280 °C for ThU. Subsequently, the reaction time was optimized over the range of 5–30 min for both sulfur sources. In addition, ligand composition was optimized by varying the OAm:oleic acid (OAc) volume ratio. For the TMS-assisted synthesis, OAm:OAc ratios of 10:0, 7:3, 5:5, and 3:7 were investigated, while for the ThU-assisted synthesis, ratios of 10:0, 7:3, 5:5, and 3:7 were also examined. Following sulfur precursor injection, the reaction mixture was maintained at the selected temperature for the predetermined duration to ensure complete nanocrystal growth. After the reaction, the solution was rapidly cooled to room temperature using an external fan. Hexane was added at approximately 100 °C to quench the reaction. The resulting nanocrystals were purified by repeated centrifugation at 6000 rpm using an ethanol/hexane mixture (1:1, v/v) as the washing solvent. Finally, the purified products were dried in air at 50 °C for 24 h.

**Characterization of CuSbS_2_ Nanocrystals**

The crystal structure of the synthesized CuSbS_2_​ nanocrystals was analyzed by X-ray diffraction (XRD) using a Bruker D8 Advance diffractometer equipped with Cu Kα radiation (λ = 1.5410λ=1.5410 Å). The optical properties of the films were evaluated using a Shimadzu UV-2600 UV–Vis spectrophotometer in the wavelength range of 400–1200 nm. The valence band position and work function were investigated by ultraviolet photoelectron spectroscopy (UPS), while the conduction band position was estimated based on the optical band gap. The surface morphology of the synthesized structures was examined by high-resolution scanning electron microscopy (HR-SEM) using a Hitachi SU-500 instrument.

**Fabrication of Perovskite Solar Cell**

For the fabrication of the electron transport layer (ETL), a diluted SnO_2_​ colloidal solution was prepared in deionized water and stirred at room temperature for 2 h. The SnO_2_ solution was then spin-coated onto fluorine-doped tin oxide (FTO) substrates at 4000 rpm for 30 s. The coated substrates were subsequently annealed at 150 °C for 30 min, followed by UV–ozone treatment for 15 min, and then immediately transferred into a nitrogen-filled glovebox. After ETL deposition, the perovskite absorber layer, hole transport layer (HTL), and metal electrode were sequentially fabricated inside the glovebox. The perovskite precursor solution was prepared by dissolving 0.05 M CsI, 0.85 M FAI, 0.10 M MABr, 0.85 M PbI_2_​, and 0.15 M PbBr_2_​ in a mixed solvent of *N,N*-dimethylformamide (DMF) and dimethyl sulfoxide (DMSO) (4:1, v/v). The perovskite films were deposited by a two-step spin-coating process at 1000 rpm for 10 s and 6000 rpm for 30 s. During the second step, 100 μL of chlorobenzene was dropped onto the spinning substrate 10 s before the end of the program to induce rapid crystallization and remove excess coordinating solvent. The deposited films were then annealed at 100 °C for 60 min. Subsequently, CuSbS_2_ nanocrystals were dispersed in chlorobenzene and deposited as the HTL. To identify the optimal HTL deposition conditions, the concentration of the CuSbS_2_​ dispersion and the spin-coating speed were systematically varied. Finally, a gold (Au) electrode with a thickness of approximately 100 nm was thermally evaporated under high vacuum (5×10^−4^ Pa) to complete the perovskite solar cell fabrication.

**Characterizations of Perovskite Solar Cell**

The surface morphology of the films was investigated by field-emission scanning electron microscopy (FE-SEM, Hitachi SU-500). Optical absorption measurements were performed using a Shimadzu UV-2600 UV–Vis spectrophotometer in the wavelength range of 300–900 nm. Steady-state photoluminescence (PL) spectra were recorded using an Edinburgh Instruments FLS920P fluorescence spectrometer with a 460 nm excitation source. The wetting behavior of the film surfaces was evaluated by time-dependent contact angle measurements (CAM) at room temperature.

SCLC measurements were carried out under dark conditions using hole-only devices with the structures FTO/SnO_2_​/Perovskite/CuSbS_2_/Au. The J–V characteristics were recorded using a Keithley 4200 Semiconductor Characterization System at a scan rate of 10 mV s^−1.^

Photovoltaic J–V measurements were performed under simulated AM 1.5G illumination using a 450 W xenon lamp (Oriel) equipped with a Schott K113 Tempax filter. The light intensity was calibrated using a certified Si reference diode (KG-3, Schott) with an infrared cut-off filter. A black metal mask was used during the measurements to define the active device area. EQE spectra were recorded using an Enli-Tech system equipped with a 300 W xenon lamp, a Si detector, and a monochromator.

Humidity stability tests were conducted under ambient conditions at 50–70% relative humidity. Light-soaking stability measurements were performed under continuous white LED illumination (1 sun equivalent) in a nitrogen atmosphere at 65 °C according to the ISOS-L-1I protocol. Thermal stability tests were carried out over 30 days, with the devices stored at 65 °C for the first 10 days and at 85 °C for the subsequent 20 days.

**Calculation of Related Parameter**

The optical bandgap (Eg) of the synthesized CuSbS_2​_ nanoparticles was estimated from the diffuse reflectance spectra using the Kubelka–Munk (K–M) approach. The Kubelka–Munk function, F(R), was calculated from the reflectance (R) using Eq. (S1):

| $F\left( R \right)= \frac{\left( 1-R \right)^{2}}{2R}$ | (S1) |
| --- | --- |

Because F(R) is proportional to the absorption coefficient (α), the optical bandgap was determined using the Tauc relation for direct allowed transitions (n=2):

| $F\left( R \right)\propto\alpha\propto= \frac{\left( h\nu-E_{g} \right)^{1/n}}{h\nu}$ | (S2) |
| --- | --- |
| ${(F\left( R \right)h\nu)}^{2}$= A$\left( h\nu- E_{g} \right)$ | (S3) |

where A is a constant and hν is the photon energy. Based on this analysis, the optical bandgap of the synthesized CuSbS_2_​ nanoparticles was determined to be 1.52 eV.


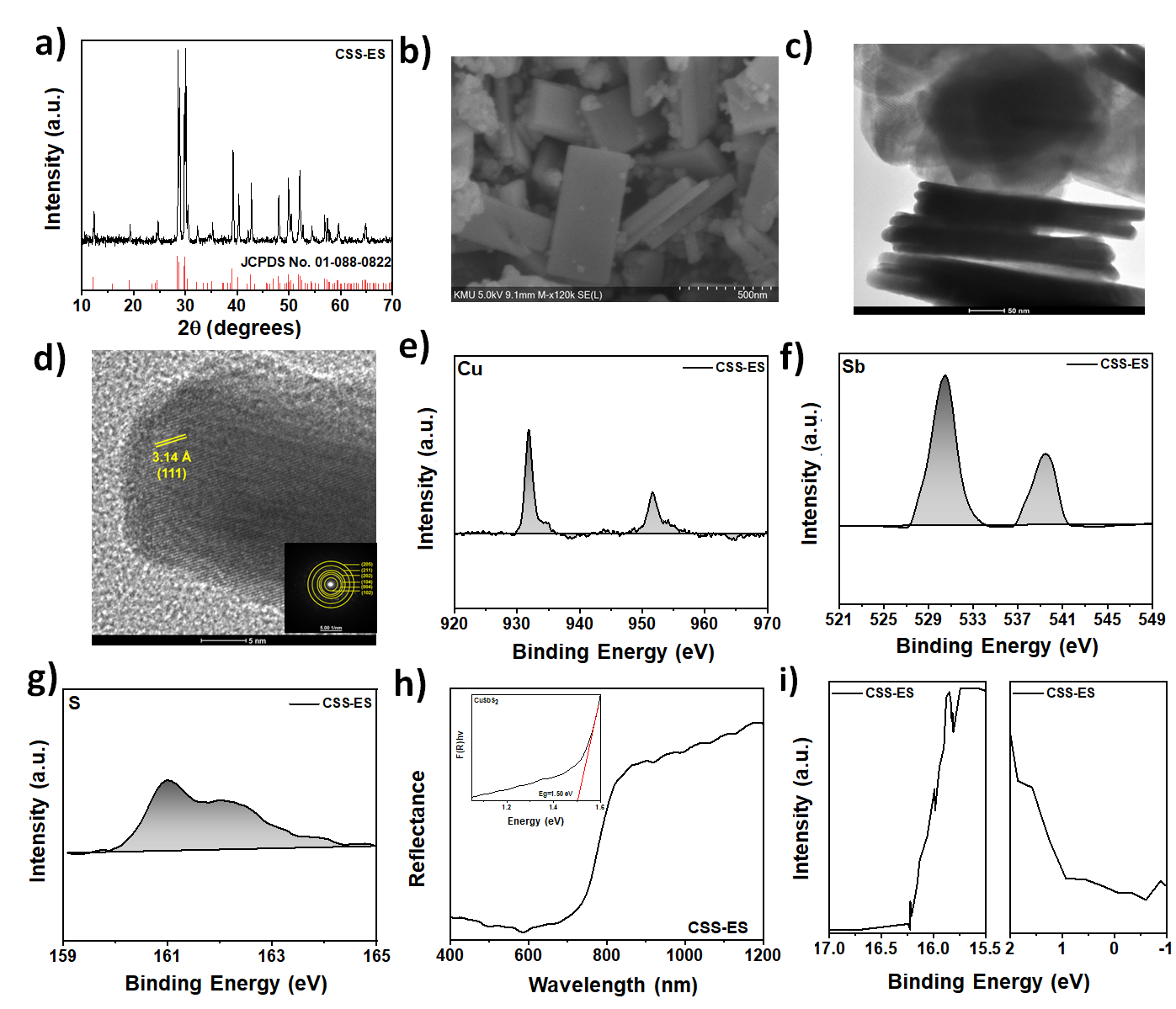


**Fig. S1.** Structural, morphological, chemical, optical, and electronic characterization of the elemental sulfur-derived CuSbS₂ reference sample (CSS-ES): (a) XRD pattern, (b) SEM image, (c) TEM image, (d) HR-TEM image with the corresponding SAED pattern. (e) High-resolution XPS spectra of Cu, (f) Sb and (g) S. (h) Diffuse reflectance spectrum with band-gap analysis in the inset. (i) UPS measurement of CSS-ES.


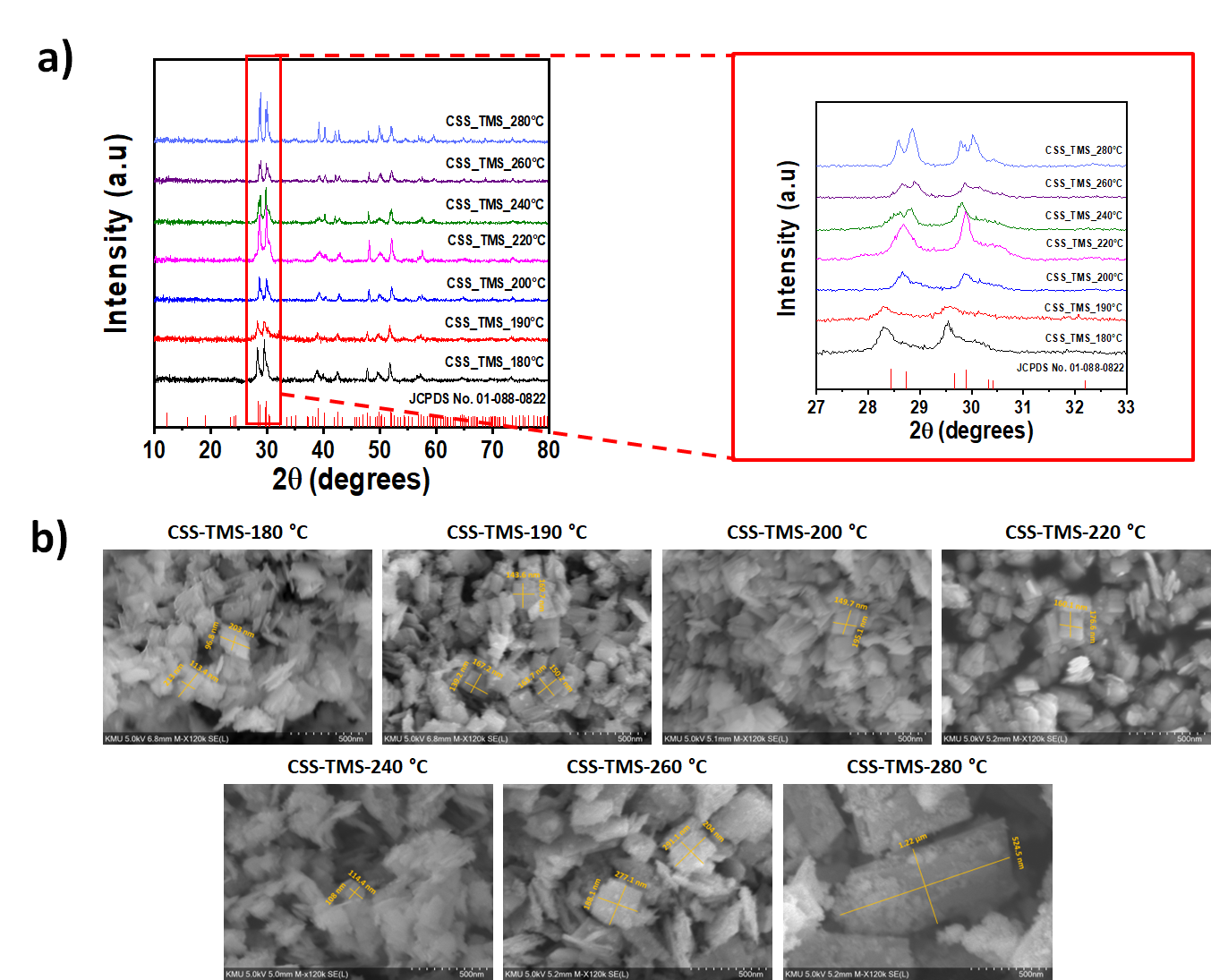


**Figure S2:** (a) X-ray diffraction (XRD) patterns of CuSbS_2_ nanoparticles synthesized via the TMS-assisted route at different reaction temperatures (180, 190, 200, 220, 240, 260, and 280 °C), with the enlarged view highlighting the evolution of the main diffraction region. (b) SEM images of the corresponding CSS-TMS samples, showing the temperature-dependent morphological evolution and the progressive development of platelet-like CuSbS_2_ nanostructures.


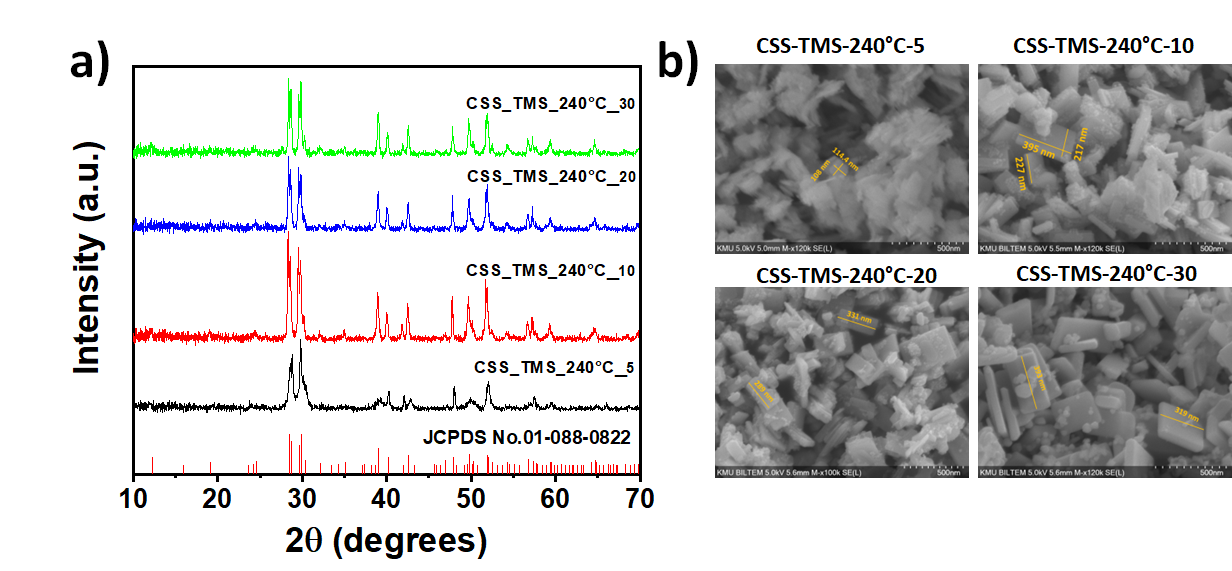


**Figure S3:** (a) XRD patterns of CuSbS₂ nanoparticles synthesized via the TMS-assisted route at 240 °C for different reaction times (5, 10, 20, and 30 min). (b) SEM images of the corresponding CSS-TMS samples, illustrating the effect of reaction time on crystallization behaviour and particle morphology.


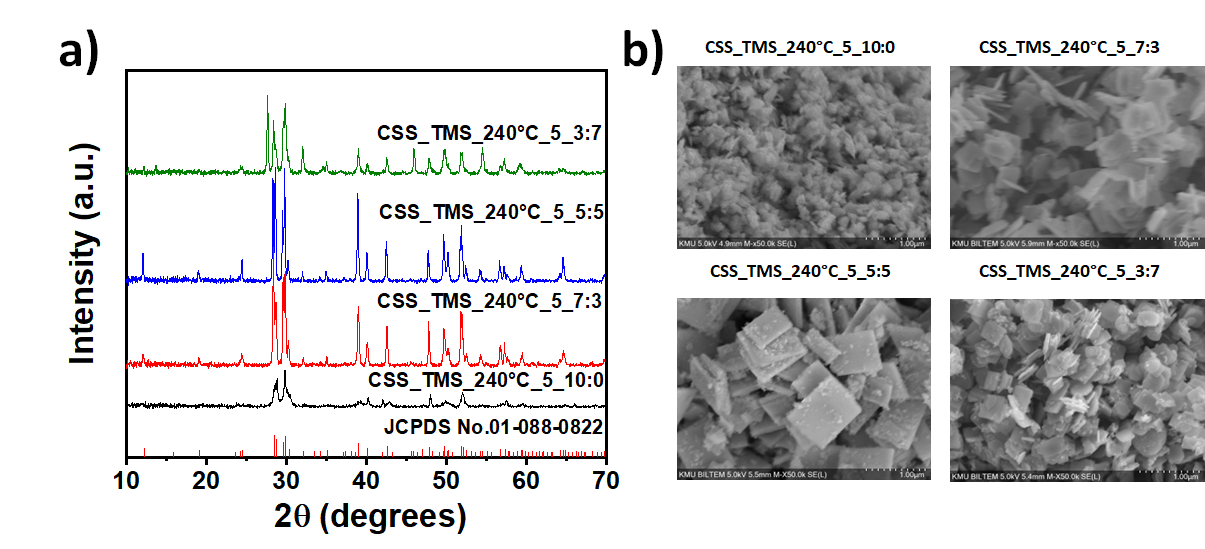


**Figure S4:** (a) XRD patterns of CuSbS_2_ nanoparticles synthesized via the TMS-assisted route at 240 °C by varying the oleylamine/oleic acid (OAm/OAc) ligand ratio (10:0, 7:3, 5:5, and 3:7). (b) SEM images of the corresponding CSS-TMS samples, showing the influence of ligand composition on phase formation, particle shape, and size distribution.


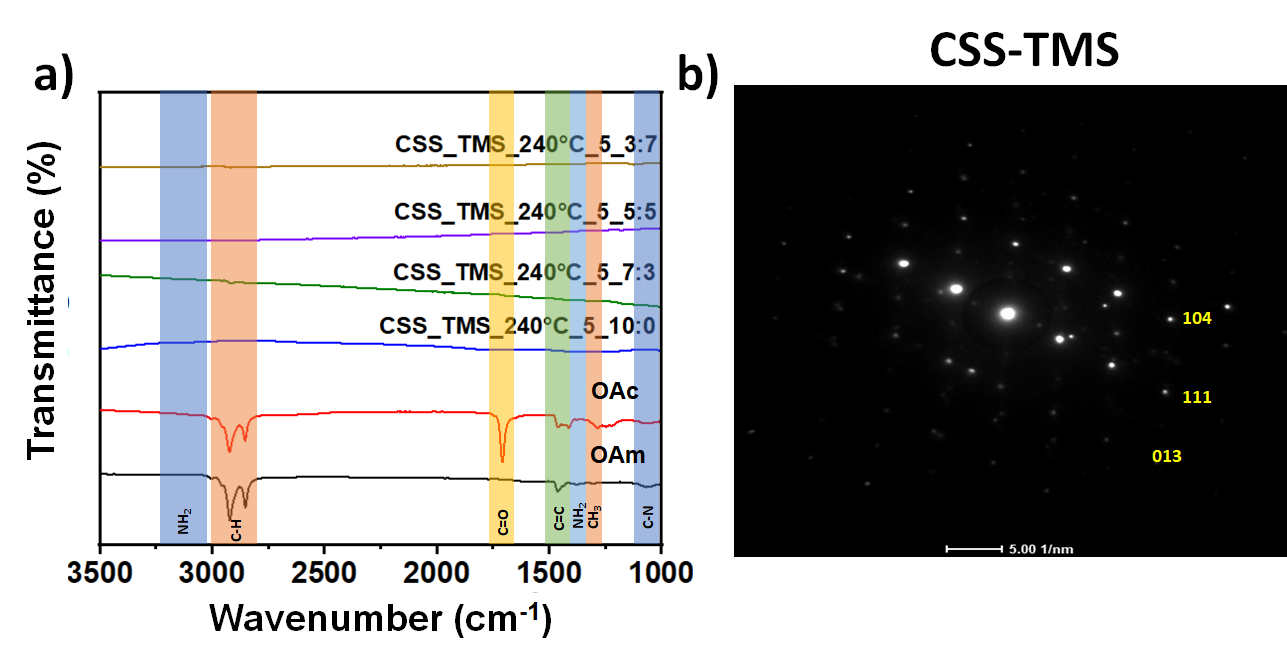


**Figure S5:** (a) FTIR spectra of CSS-TMS samples synthesized under different OAm/OAc ligand ratios, together with reference spectra of oleylamine (OAm) and oleic acid (OAc), showing the characteristic surface-bound ligand functionalities. (b) SAED pattern of the optimized CSS-TMS sample, confirming the crystalline nature of the CuSbS_2_ nanostructure.


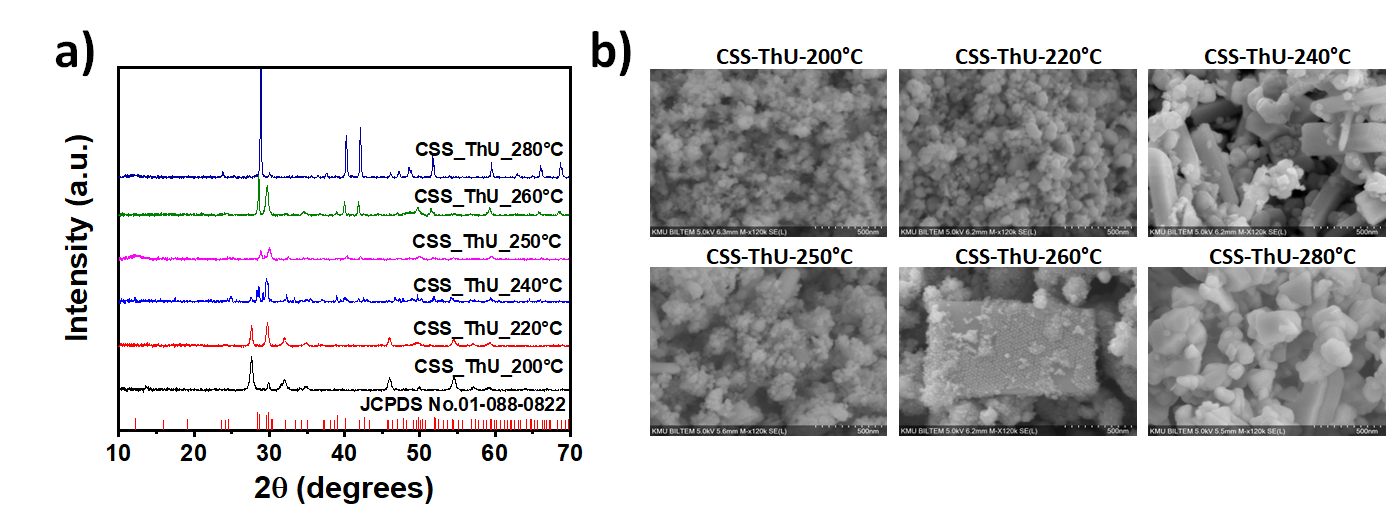


**Figure S6:** (a) XRD patterns of CuSbS_2_ nanoparticles synthesized via the thiourea-assisted route at different reaction temperatures (200, 220, 240, 250, 260, and 280 °C). (b) SEM images of the corresponding CSS-ThU samples, showing the temperature-dependent evolution of particle morphology and the formation of mixed plate-like and quasi-spherical CuSbS_2_ nanostructures.


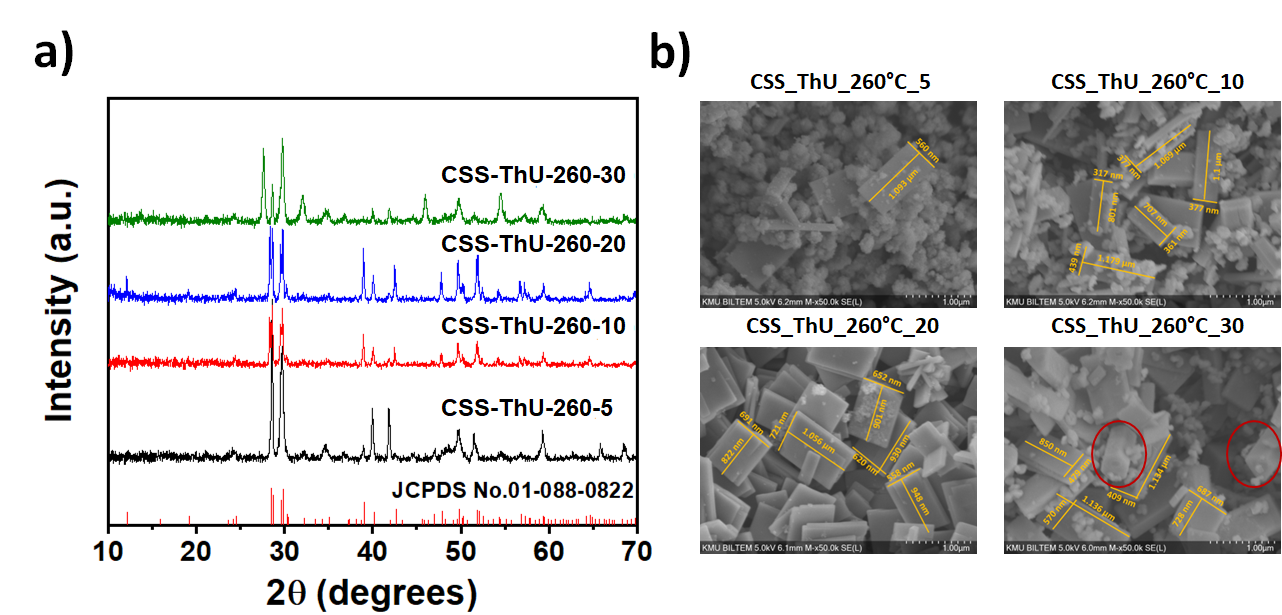


**Figure S7:** (a) XRD patterns of CuSbS_2_ nanoparticles synthesized via the thiourea-assisted route at 260 °C for different reaction times (5, 10, 20, and 30 min). (b) SEM images of the corresponding CSS-ThU samples, illustrating the influence of reaction time on crystal growth, particle development, and morphological uniformity.


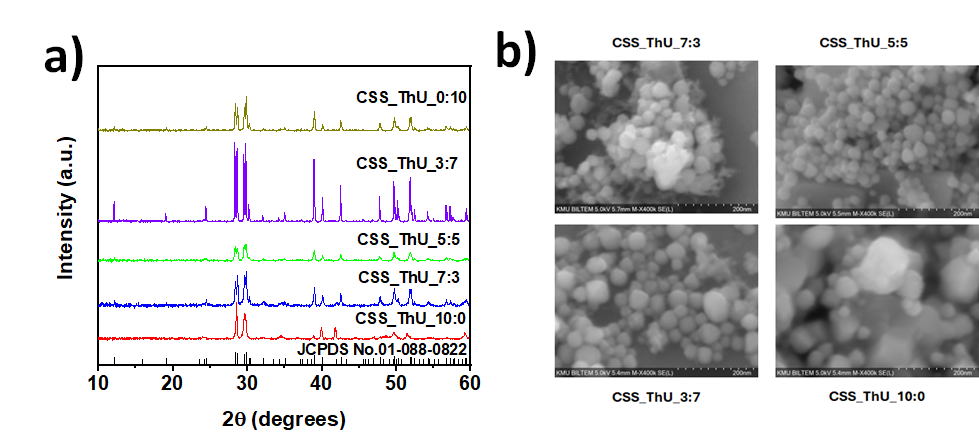


**Figure S8:** (a) XRD patterns of CuSbS_2_ nanoparticles synthesized via the thiourea-assisted route under different oleylamine/oleic acid (OAm/OAc) ligand ratios (10:0, 7:3, 5:5, and 3:7). (b) SEM images of the corresponding CSS-ThU samples, highlighting the ligand-ratio-dependent changes in particle distribution and the formation of quasi-spherical CuSbS_2_ nanostructures.


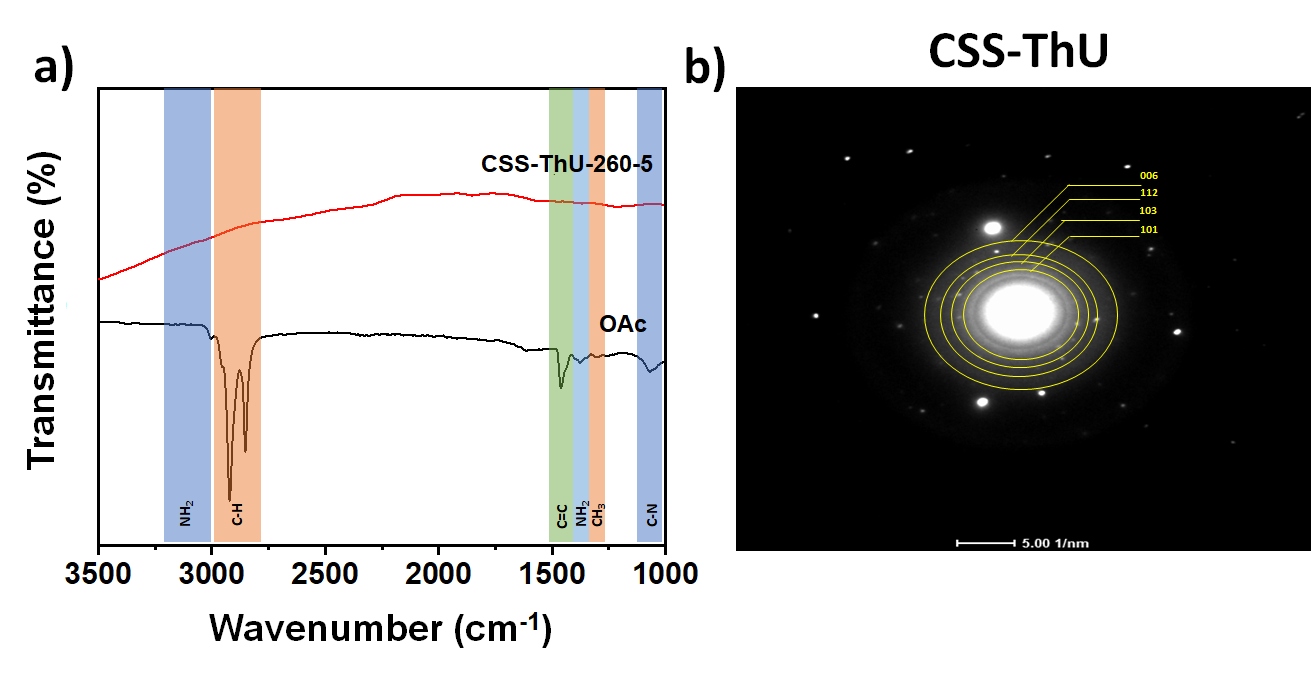


**Figure S9:** (a) FTIR spectrum of the optimized CSS-ThU sample together with the reference spectrum of oleylamine (OAc), confirming the presence of ligand-derived surface functionalities. (b) SAED pattern of the optimized CSS-ThU sample, indicating the polycrystalline yet highly crystalline nature of the thiourea-derived CuSbS_2_ nanostructure.


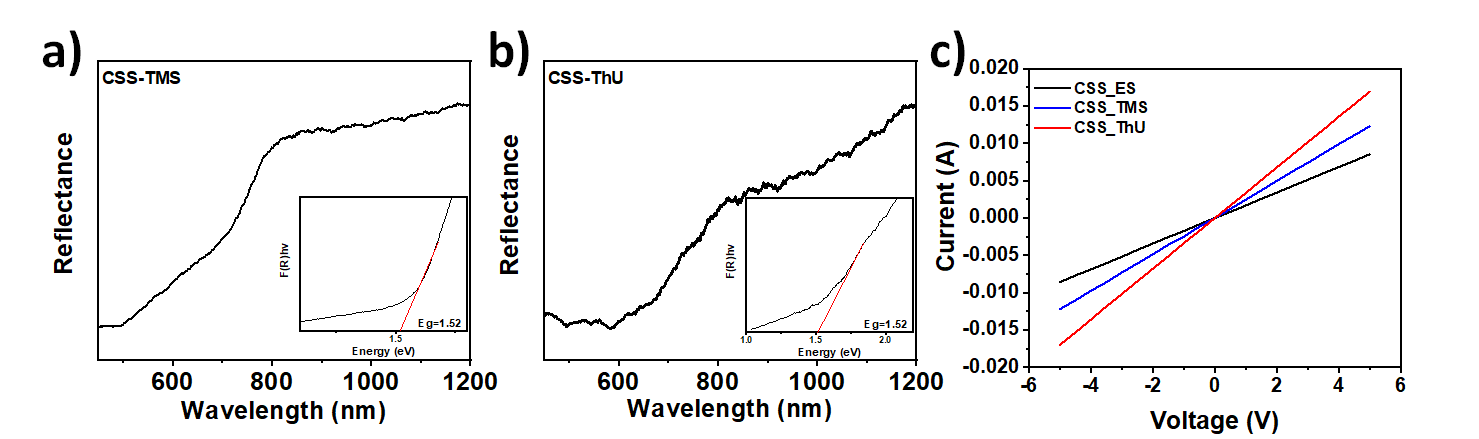


**Figure S10:** (a) Reflectance spectrum of CSS-TMS and the corresponding optical bandgap estimation, (b) Reflectance spectrum of CSS-ThU and the corresponding optical bandgap estimation, and (c) Current–voltage (*I–V*) characteristics of optimized CuSbS_2_ samples synthesized using ES(CSS-ES), TMS (CSS-TMS) and thiourea (CSS-ThU), demonstrating the steeper slope and improved electrical conductivity of the thiourea-derived sample.


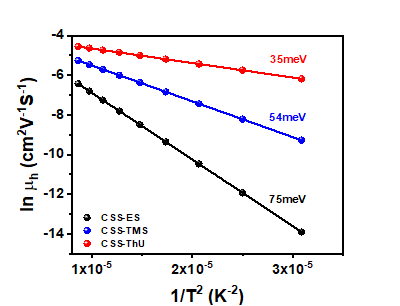


**Figure S11:** Temperature-dependent hole mobility analysis of CSS-ES, CSS-TMS, and CSS-ThU HTLs plotted as ln(μT) versus 1/T² according to the Gaussian disorder model.


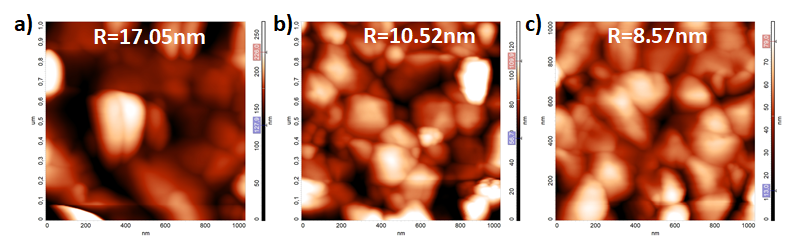


**Figure S12:** AFM height images and corresponding root-mean-square (RMS) surface roughness values of perovskite films coated with (a) CSS-ES, (b) CSS-TMS, and (c) CSS-ThU hole-transport layers.


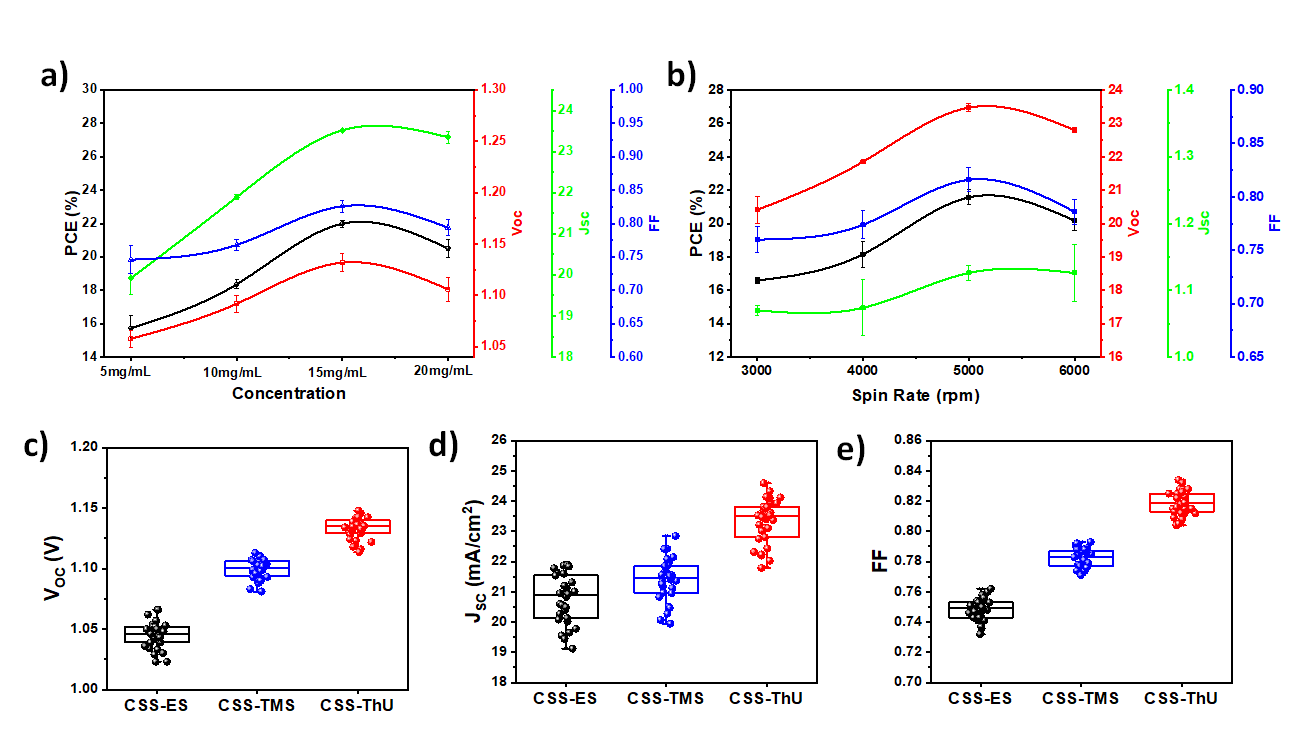


**Figure S13:** Optimization of the CSS-ThU hole-transport layer deposition conditions in perovskite solar cells. (a) Effect of CuSbS_2_ concentration on photovoltaic parameters (PCE, *Voc*​, *Jsc*​, and *FF*). (b) Effect of spin-coating speed on photovoltaic parameters (PCE, *Voc*​, *Jsc*, and *FF*). Statistical box plots of c) *Voc*​, d) *Jsc​*, and e) *FF*, respectively, obtained from 30 independent devices using CSS-ES, CSS-TMS, and CSS-ThU HTLs.


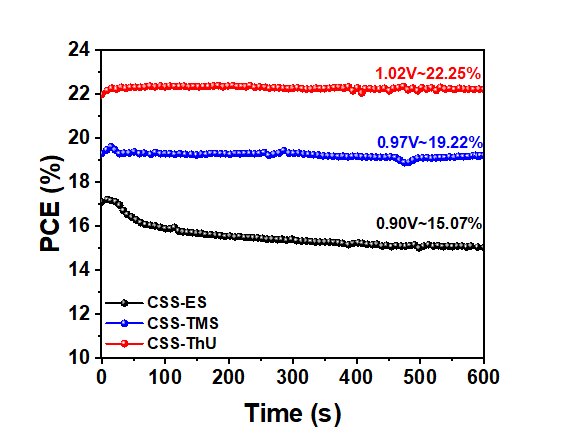


**Figure S14:** Steady-state power output (SPO) measurements of perovskite solar cells employing CSS-ES, CSS-TMS, and CSS-ThU as hole-transport layers, recorded under continuous illumination at their respective maximum power point conditions.


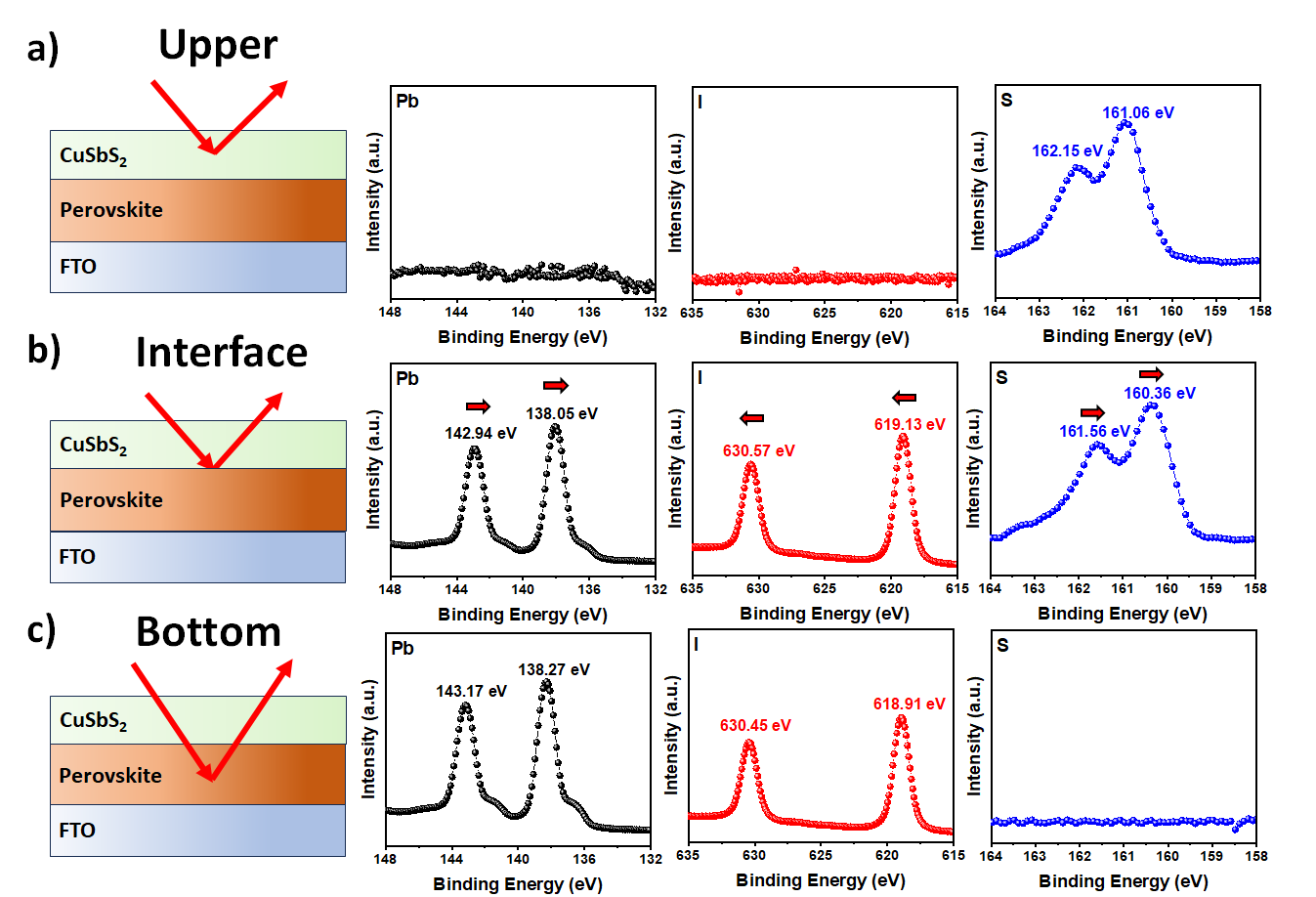


**Figure S15:** Depth-dependent XPS characterization of the CuSbS₂/perovskite heterointerface. High-resolution XPS spectra of Pb 4f, I 3d, and S 2p were collected from corresponding cross-section of the device at three representative regions: (a) the upper CuSbS₂ surface, (b) the CuSbS₂/perovskite interface, and (c) the bottom perovskite layer.


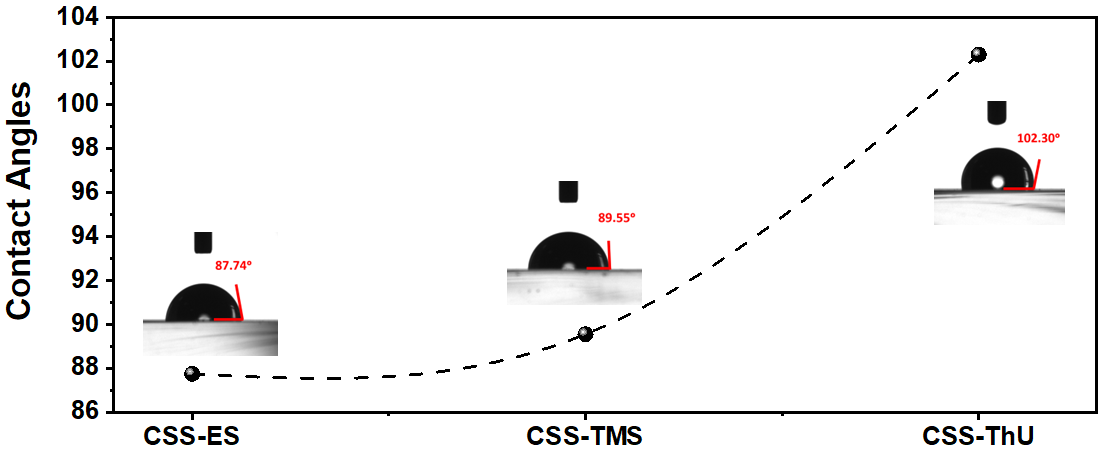


**Figure S16:** Static water contact angle measurements of CSS-ES, CSS-TMS, and CSS-ThU films.

**Table S1:** Literature summary of chalcogenide-based hole transport layers (HTLs) used in perovskite solar cells and their corresponding photovoltaic performance parameters.

| **HTL Material** | **Type** | **Device Architecture** | **Voc**  **(V)** | **Jsc**  **(mA cm^-2^)** | **FF** | **PCE**  **(%)** | **Light** | **Moisture** | **Thermal** | **Ref** |
| --- | --- | --- | --- | --- | --- | --- | --- | --- | --- | --- |
| Cu_12_Sb_4_S_13_ | n-i-p | FTO/C-TiO_2_/m-TiO_2_/MAPbI_3_/Cu_12_Sb_4_S_13_/Au | 0.80 | 18.08 | 0.45 | 6.5 | ~50% 15 days ~25°C | - | - | [1] |
| Cu_12_Sb_4_S_13_ | n-i-p | FTO/C-TiO_2_/m-TiO_2_/MAPbI_3_/Cu_12_Sb_4_S_13_/Au | 1.06 | 22.50 | 0.67 | 15.43 | - | ~94% 1000h (ambient air) | - | [2] |
| Cu_12_Sb_4_S_13_ | n-i-p | FTO/C-TiO_2_/m-TiO_2_/MAPbI_3_/Cu_12_Sb_4_S_13_/Au | 1.05 | 21.85 | 0.61 | 14.13 | - | ~91% 30 days 40% RH and 25 °C | - | [3] |
| Cu_12_Sb_4_S_13_ | n-i-p | FTO/C-TiO_2_/m-TiO_2_/CsPbI_3_/Cu_12_Sb_4_S_13_/Au | 1.04 | 18.28 | 0.52 | 10.02 | - | ~94% 360h (ambient air) | - | [4] |
| CuSbS_2_ | n-i-p | FTO/C-TiO_2_/m-TiO_2_/CsPbI_3_/CuSbS_2_+Nb_2_O_5_/Au | 1.21 | 11.86 | 0.67 | 9.11 | - | ~98% 20h (ambient air) | - | [5] |
| Cu_3_SbS_4_ | n-i-p | FTO/C-TiO_2_/m-TiO_2_/ Cs_0.05_ (MA_0.17_FA_0.83_)_0.95_ Pb (I_0.83_Br_0.17_)_3_/ Cu_3_SbS_4_/Au | 1.01 | 18.84 | 0.68 | 6.66 | - | ~75% 90 days 50% RH and ~25°C | - | [6] |
| Cu_2_SnS_2_ | n-i-p | FTO/c-mp TiO_2_/ Cs_0.05_ (MA_0.17_FA_0.83_)_0.95_ Pb (I_0.83_Br_0.17_)_3_/Cu_2_SnS_3_/Au | 1.06 | 20.59 | 0.69 | 13.01 | - | ~90% 1200h 40% RH and 25 °C | - | [7] |
| Cu_2_SnS_3_ | n-i-p | ITO/SnO_2_/ FA_0.87_MA_0.13_PbI_2.87_Br_0.13_/ Cu_2_SnS_3_/Au | 0.97 | 22.91 | 0.71 | 15.96 | - | ~94% 9 days 80% RH and 25 °C | - | [8] |
| Cu_2_SnS_3_ | n-i-p | FTO/SnO_2_/FAPbI_3_/Cu_2_SnS_3_/C | 1.05 | 23.71 | 0.67 | 16.55 | ~100% 30 days ~25°C (dry air) | - | - | [9] |
| Cu_2-x_ GeS_3_ | n-i-p | FTO/c-TiO_2_/MAPbI_3_/Cu_2-x_ GeS_3_/Au | 1.06 | 18.55 | 0.63 | 12.56 | - | 95% 30 days 50% RH and ~25°C | - | [10] |
| CuFeS_2_ | n-i-p | ITO/SnO_2_/ FA_0.87_MA_0.13_PbI_2.87_Br_0.13_/ CuFeS_2_/Au | 1.11 | 23.86 | 0.76 | 20.17 | - | 93% 30 days 30% RH and 25°C | - | [11] |
| CuGaS_2_ | n-i-p | ITO/SnO_2_/ FA_0.85_MA_0.15_Pb(I_0.85_Br_0.15_)_3_/CuGaS_2_/Au | 1.05 | 22.89 | 0.70 | 17.34 | - | ~87% 500 h 40% RH and 25 °C | - | [12] |
| CuGaS_2_ | n-i-p | FTO/c-TiO_2_/ (FAI)_0.81_(PbI_2_)_0.85_ (MAPbBr_3_)_0.15_CuGaS_2_/Au | 1.06 | 18.79 | 0.64 | 12.78 | - | - | - | [13] |
| CuGaS_2_ | n-i-p | FTO/c-mp TiO_2_/Cs_0.05_ (MA_0.17_FA_0.83_)_0.95_ Pb (I_0.83_Br_0.17_)_3_/CuGaS_2_/Au | 1.07 | 22.08 | 0.59 | 13.92 | - | ~95% 30 days ~40% RH and ~25°C | - | [14] |
| CuInS_2_ | n-i-p | ITO/SnO_2_/ FA_0.87_MA_0.13_PbI_2.87_Br_0.13_/ CuInS_2_/Au | 1.06 | 22.58 | 0.67 | 16.13 | - | 95% 32 days 40% RH and ~25°C | - | [15] |
| CuInS_2_ | n-i-p | ITO/SnO_2_/ FA_0.87_MA_0.13_PbI_2.87_Br_0.13_/ CuInS_2_/Spiro-OmetaD/Au | 1.08 | 23.25 | 0.76 | 19.05 | - | ~91% 30 days 40% RH and 25 °C | - | [16] |
| CuInS_2_ | n-i-p | FTO/c-TiO_2_/m-TiO_2_/ Cs_0.05_ (MA_0.17_FA_0.83_)_0.95_ Pb (I_0.83_Br_0.17_)_3_/CuInS_2_/PTAA/C | 1.08 | 20.99 | 0.69 | 16 | - | ~100% 1720 h 15% RH and dark 25 °C | ~70% 405 h 40% RH and 60 °C | [17] |
| CuInS_2_ | n-i-p | ITO/SnO_2_/MAPbI_3_/CuInS_2_/Au | 1.07 | 23.50 | 0.75 | 18.8 | - | 91% 30 days 20% RH and ~25°C | - | [18] |
| CuInS_2_ | n-i-p | ITO/SnO_2_/MAPbI_3_/CuInS_2_/Au | 0.98 | 24.54 | 0.9 | 18.8 | - | 94.3% 10 days 20% RH and ~25°C | - | [19] |
| CuInS_2_ | n-i-p | FTO/c-mp TiO_2_/ Cs_0.05_ (MA_0.17_FA_0.83_)_0.95_ Pb (I_0.83_Br_0.17_)_3_/CuInS_2_/C | 1.03 | 23.98 | 0.96 | 17.65 | - | - | - | [20] |
| CuInS_2_ | n-i-p | FTO/c-mp TiO_2_/MAPbI_3_/CuInS_2_/C | 1.06 | 24.29 | 0.63 | 16.11 | - | 92% 4000h (RH 45%, ~25°C) | - | [21] |
| CuInS_2_ | n-i-p | ITO/SnO_2_/MAPbI_3_/CuInS_2_/Au | 0.86 | 22.5 | 0.55 | 12.8 | - | 78% 96h 40% RH and ~25°C | - | [22] |
| CuInS_2_ | p-i-n | ITO/CuInS_2_/Al_2_O_3_/MAPbI_3_:CdS/PC60BM/Ag | 0.95 | 23.8 | 0.73 | 16.5 | - | - | - | [23] |
| CuInS_2_ | p-i-n | ITO/CuInS_2_/MAPbI_3_/PC60BM/BCP/Ag | 0.92 | 23 | 0.82 | 17.34 | - | 70% 96h 30% RH and 25°C | - | [24] |
| CuSbS_2_ | n-i-p | FTO/SnO_2_/ Cs_0.05_ FA_0.85_MA_0.1_Pb(I_0.85_Br_0.15_)_3_/  CuSbS_2_/ Au | 1.15 | 24.33 | 0.82 | 22.72 | ~56% 1000 h  100 mW cm⁻² LED, N₂, 65 °C | ~80% 1000 h  50–70% RH, 25 °C, dark | ~78% 30 d  N₂, 65 °C (10 d) / 85 °C (20 d) | This Work |

**REFERENCES**

(1) Tamilselvan, M.; Bhattacharyya, A. J. Tetrahedrite (Cu_12_Sb_4_S_13_) ternary inorganic hole conductor for ambient processed stable perovskite solar cells. *ACS Applied Energy Materials* **2018**, *1* (8), 4227-4234.

(2) Long, C.; Peng, Z.; Huang, J.; Wang, Y.; Luo, W.; Fu, Y.; Chen, J.; Chen, J. Enhancement on charge transfer properties of Cu_12_Sb_4_S_13_ quantum dots hole transport materials by surface ligand modulation in perovskite solar cells. *New Journal of Chemistry* **2022**, *46* (24), 11751-11758.

(3) Liu, Y.; Chen, Q.; Mei, A.; Hu, B.; Yang, Z.; Chen, W. Bandgap aligned Cu_12_Sb_4_S_13_ quantum dots as efficient inorganic hole transport materials in planar perovskite solar cells with enhanced stability. *Sustainable Energy & Fuels* **2019**, *3* (3), 831-840.

(4) Liu, Y.; Zhao, X.; Yang, Z.; Li, Q.; Wei, W.; Hu, B.; Chen, W. Cu_12_Sb_4_S_13_ quantum dots with ligand exchange as hole transport materials in all-inorganic perovskite CsPbI_3_ quantum dot solar cells. *ACS Applied Energy Materials* **2020**, *3* (4), 3521-3529.

(5) Mujtaba, A.; Khan, M.; Albarzan, B.; Alotaibi, N.; Almutairi, B. S. Reduced Recombination for Achieving Improved Efficiency of CsPbIBr_2_ PSCs using SnO_2_ and Nb_2_O_5_-substituted CuSbS_2_ as Hole Transport Layers. *Physica B: Condensed Matter* **2026**, 418327.

(6) Mohamadkhani, F.; Heidariramsheh, M.; Javadpour, S.; Ghavaminia, E.; Mahdavi, S. M.; Taghavinia, N. Sb_2_S_3_ and Cu_3_SbS_4_ nanocrystals as inorganic hole transporting materials in perovskite solar cells. *Solar Energy* **2021**, *223*, 106-112.

(7) Heidariramsheh, M.; Mirhosseini, M.; Abdizadeh, K.; Mahdavi, S. M.; Taghavinia, N. Evaluating Cu_2_SnS_3_ nanoparticle layers as hole-transporting materials in perovskite solar cells. *ACS Applied Energy Materials* **2021**, *4* (6), 5560-5573.

(8) Ma, M.; Zhou, Q.; Ma, W.; Zhang, Z.; Kang, M.; Gao, W.; Ma, X.; Liu, Y.; Mao, Y. Alloyed (Cu_2_SnS_3_)_x_(ZnS)_1−x_ quantum dots as a hole-transporting layer for efficient and stable perovskite solar cells. *Solar Energy* **2021**, *224*, 1170-1177.

(9) Yu, Z.; Li, W.; Cheng, N.; Liu, Z.; Lei, B.; Xiao, Z.; Zi, W.; Zhao, Z.; Tu, Y. Cu2SnS3 nanocrystal-based hole-transport layer for carbon electrode-based perovskite solar cells. *ACS Applied Nano Materials* **2022**, *5* (8), 10755-10762.

(10) Jin, X.; Lei, X.; Wu, C.; Jiang, G.; Liu, W.; Zeng, H.; Chen, T.; Zhu, C. Cu_2−x_GeS_3_: A new hole transporting material for stable and efficient perovskite solar cells. *Journal of Materials Chemistry A* **2017**, *5* (37), 19884-19891.

(11) Xiao, S.-Q.; Liu, L.-Z.; Zhang, Z.-L.; Liu, Y.-F.; Gao, H.-P.; Zhang, H.-F.; Mao, Y.-L. Surface modification with CuFeS_2_ nanocrystals to improve the efficiency and stability of perovskite solar cells. *ACS Applied Materials & Interfaces* **2023**, *15* (24), 29178-29185.

(12) Ma, W.; Zhang, Z.; Ma, M.; Liu, Y.; Pan, G.; Gao, H.; Mao, Y. CuGaS_2_ quantum dots with controlled surface defects as an hole-transport material for high-efficient and stable perovskite solar cells. *Solar Energy* **2020**, *211*, 55-61.

(13) Wang, X.; Kou, D.; Ouyang, C.; Liu, J. New strategy for improving the perovskite solar cells’ open-circuit voltage: cation substitution of hole transport layer. *Optical Materials* **2021**, *121*, 111262.

(14) Forouzandeh, M.; Behrouznejad, F.; Ghavaminia, E.; Khosroshahi, R.; Li, X.; Zhan, Y.; Liao, Y.; Ning, Z.; Taghavinia, N. Effect of indium ratio in CuIn_x_Ga_1-x_S_2_/carbon hole collecting electrode for perovskite solar cells. *Journal of Power Sources* **2020**, *475*, 228658.

(15) Zhang, Z.; Ma, W.; Zhou, Q.; Mao, Y. Double inorganic hole extraction layer of Cs: NiO_x_/CuInS_2_ for efficiency and stability enhancement of perovskite solar cells. *International Journal of Energy Research* **2022**, *46* (10), 13908-13914.

(16) Zhou, Q.; Ma, W.; Zhang, Z.; Liu, Y.; Zhang, H.; Mao, Y. Double-layered hole transport material of CuInS_2_/Spiro for highly efficient and stable perovskite solar cells. *Organic Electronics* **2021**, *96*, 106249.

(17) Kassem, H.; Salehi, A.; Kahrizi, M.; Jamali, Z. CuInS_2_/Poly (triarylamine)(PTAA) binary composite as an efficient hole transporter for carbon electrode-based perovskite solar cells. *Materials Research Bulletin* **2024**, *170*, 112557.

(18) Liu, Y.; Zhang, Z.; Gao, H.; Zhang, H.; Mao, Y. A novel inorganic hole-transporting material of CuInS_2_ for perovskite solar cells with high efficiency and improved stability. *Organic Electronics* **2019**, *75*, 105430.

(19) Ahmed, R. Rebirth of CuInS_2_ as hole transport material for perovskite solar cells. *SmartMat* **2023**, *4* (6), e1195.

(20) Ghavaminia, E.; Behrouznejad, F.; Forouzandeh, M.; Khosroshahi, R.; Darbari, S.; Zhan, Y.; Taghavinia, N. Polyvinylcarbazole as an Efficient Interfacial Modifier for Low‐Cost Perovskite Solar Cells with CuInS_2_/Carbon Hole‐Collecting Electrode. *Solar RRL* **2021**, *5* (7), 2100074.

(21) Heydari, M.; Mohammadi, M.; Baghestani, E.; Tajabadi, F.; Bowman, A. R.; Roose, B.; Forouzandeh, M.; Heidariramsheh, M.; Stranks, S. D.; Abdi, Y. Charged defect healing by N, N′–di (naphthalene-1-yl)-N, N′ diphenyl benzidine at the interface of CuInS_2_ nanoparticle hole transporting materials in carbon-based halide perovskite solar cells. *Journal of Power Sources* **2023**, *581*, 233498.

(22) Zhang, Y.; Zhang, Z.; Liu, Y.; Liu, Y.; Gao, H.; Mao, Y. An inorganic hole-transport material of CuInSe_2_ for stable and efficient perovskite solar cells. *Organic Electronics* **2019**, *67*, 168-174.

(23) Chen, C.; Zhai, Y.; Li, F.; Tan, F.; Yue, G.; Zhang, W.; Wang, M. High efficiency CH_3_NH_3_PbI_3_: CdS perovskite solar cells with CuInS_2_ as the hole transporting layer. *Journal of Power Sources* **2017**, *341*, 396-403.

(24) Yang, S.; Ma, W.; Zhang, Z.; Zhu, J.; Liu, Y.; Zhang, H.; Mao, Y. Inverted perovskite solar cells based on inorganic hole transport material of CuInS_2_ with high efficiency and stability. *Solar Energy* **2021**, *230*, 485-491.
